# Single-cell heterogeneity of adipose eosinophils is shaped by sex and RELMα and linked to macrophage communication networks in obesity

**DOI:** 10.1093/pnasnexus/pgag365

**Published:** 2026-10-24

**Authors:** Rebecca E Ruggiero-Ruff, Xinru Qiu, Lauren Vance, Yuxin He, Djurdjica Coss, Adam Godzik, Meera G Nair

**Affiliations:** Division of Biomedical Sciences, School of Medicine, University of California, Riverside, Riverside, CA 92521, USA; Division of Biomedical Sciences, School of Medicine, University of California, Riverside, Riverside, CA 92521, USA; Division of Biomedical Sciences, School of Medicine, University of California, Riverside, Riverside, CA 92521, USA; Department of Biochemistry, Graduate Program in Biochemistry and Molecular Biology, College of Natural and Agricultural Sciences, University of California, Riverside, Riverside, CA CA92521, USA; Division of Biomedical Sciences, School of Medicine, University of California, Riverside, Riverside, CA 92521, USA; Division of Biomedical Sciences, School of Medicine, University of California, Riverside, Riverside, CA 92521, USA; Division of Biomedical Sciences, School of Medicine, University of California, Riverside, Riverside, CA 92521, USA

**Keywords:** obesity, eosinophil, macrophage, sexual dimorphism

## Abstract

Eosinophils are immune cells with terminal effector functions in type 2 immune responses, yet recent findings suggest that they exhibit functional plasticity and heterogeneity dependent on tissue-specific cues. Building on our prior functional study showing that the immunoregulatory protein RELMα and eosinophils contribute to female protection from high-fat diet (HFD)-induced obesity, we used single-cell transcriptional profiling to study adipose eosinophil states at higher resolution. We identify six transcriptionally distinct eosinophil clusters whose relative abundance differs by sex, helminth infection, and RELMα status. Adipose tissue from females or helminth-infected mice is enriched for eosinophil clusters with type 2/homeostatic-associated transcriptional programs, whereas males and RELMα-deficient mice have increased representation of inflammatory/MHCII-associated clusters together with an altered predicted macrophage–eosinophil communication networks. Spectral flow cytometry and adipose tissue in situ hybridization independently support eosinophil heterogeneity and candidate chemokine-receptor interactions. Through single-cell regulatory network inference and clustering analysis, we further identified transcription factor regulons unique to the specific eosinophil clusters, including sex-differential and HFD-enriched regulatory programs supported by target-gene expression patterns. Collectively, these data provide a framework for describing adipose eosinophil heterogeneity within a previously established RELMα–macrophage–eosinophil axis and identify candidate transcriptional and communication programs for future functional testing.

Significance Statement Prior functional work showed that RELMα and eosinophils contribute to female protection from adipose inflammation in diet-induced obesity. Here, single-cell profiling provides higher resolution, showing six adipose eosinophil clusters whose abundance shifts with helminth infection, sex, and expression of the immunoregulatory protein RELMα. Female adipose tissue is enriched in eosinophil clusters with type 2/homeostatic-associated transcriptional programs, whereas males and RELMα-deficient mice show greater representation of inflammatory/MHCII-associated clusters together with altered predicted macrophage–eosinophil communication networks. These findings identify differences between male and female adipose eosinophils in obesity and suggest a framework for testing whether terminally differentiated immune cells are remodeled across tissue environments.

## Introduction

Eosinophils are terminally differentiated cells (TDCs), known mostly for their association with many diseases, particularly type 2 immune disorders. In severe asthma, eosinophilic esophagitis (EoE), and hypereosinophilic syndromes, eosinophils release highly potent, cytotoxic granule proteins (e.g. major basic protein, eosinophil peroxidase [EPX], and eosinophil cationic protein) that cause inflammation and significant tissue damage (1–5). Recent studies highlight their role in maintaining tissue homeostasis (6–10) where they are present within the tissues under noninflamed conditions with protective functions such as preventing obesity (11–16).

Existence of different functional states in neutrophils, platelets, and eosinophils have been reported in the literature (17–21). Among many unanswered questions is whether reproducible transcriptional states of TDC exist within the tissues and what is the timeframe of their switching to different states by interactions with other cells or environmental cues. The concept of reproducible transcriptional states would fundamentally revise the paradigm that eosinophils, given their terminal differentiation status, remain homogenous in their function with little transcriptional flexibility. Such states have been identified in other TDCs such as neutrophils (22) or platelets (20) together with mechanisms driving their heterogeneity such as limited chromatin remodeling (neutrophils) or turning on/off cellular pathways (platelets). Importantly, terminal differentiation defined by exit from the cell cycle and commitment to a lineage fate does not preclude transcriptional plasticity or context-dependent functional reprogramming. However, the broader topic of how TDCs develop different functional profiles and whether this heterogeneity impacts the tissue inflammatory state remains unclear.

Eosinophils remain the least studied among immune cells with terminal effector functions because of technical difficulties in isolation, low viability, and low frequency within tissues in the absence of type 2 immune perturbations such as helminth infection and allergy (23). Bulk transcriptomic analyses of eosinophils across diverse tissue types, activation states and disease contexts have revealed that these traditionally type-2-associated terminal effector granulocytes exhibit heterogeneous and context-dependent gene expression programs that reflect distinct functional specializations (3, 9, 10, 24–29). Single-cell transcriptomics studies are beginning to resolve eosinophil heterogeneity at higher resolution and in defined activation states using transgenic models or models of type 2 inflammation to induce eosinophilia (2, 21, 23) or in tissue homeostasis within the small intestine (10, 30). However, single-cell analysis of endogenous eosinophils in the adipose tissue during obesity has not yet been reported.

To address this gap, we optimized eosinophil enrichment methodologies and single-cell fixed RNA profiling technologies to investigate eosinophil heterogeneity within the adipose tissue and determine whether they are changed in obesity in males or females. This study builds on our prior work in the same diet-induced obesity mouse model, which showed that adipose RELMα is higher in females and that RELMα deficiency causes eosinophil loss and inflammatory macrophage accumulation, which is rescued by recombinant RELMα treatment or eosinophil transfer (12). In this study, we elucidate the organizational architecture of our previously established RELMα–macrophage–eosinophil axis at a single-cell resolution. Specifically, we identify and characterize the diversity of adipose tissue eosinophil clusters and determine how their composition shifts with sex, RELMα status and helminth infection, while identifying the transcriptional and intercellular communication programs that are associated with these changes.

## Results

### Distinct transcriptional programs in adipose eosinophils in protective vs. inflammatory outcomes of obesity

We previously showed that female mice were protected from high-fat diet (HFD)-induced obesity compared to males, and that the underlying immune mechanism involved increased RELMα expression that contributed to a higher frequency of adipose eosinophils (12). To identify sex and RELMα-dependent transcriptional differences in eosinophils, single-cell RNA sequencing (scRNA-seq) was conducted on SiglecF-enriched adipose eosinophils from HFD-fed wild-type (WT) male and female mice compared to RELMα^−/−^ (KO) mice (Figs. 1A, S1, and S2). In addition, helminth-challenged HFD-fed female mice were included to evaluate how type 2 inflammation modulates eosinophil heterogeneity in the adipose tissue. scRNA-seq analysis with dimension reduction and uniform manifold approximation and projection (UMAP) identified 37,334 total cells amongst all groups, including seven immune cell types (Fig. 1B and C), including a transcriptionally distinct eosinophil cluster expressing canonical markers *Siglecf*, *Il5ra*, and *Ccr3*. Macrophages were the largest immune cell cluster (59.2%) followed by eosinophils (29.8%) despite higher eosinophil frequency prior to fixation, potentially due to eosinophil loss during the fixation through degranulation and cell death.

**Figure 1:**
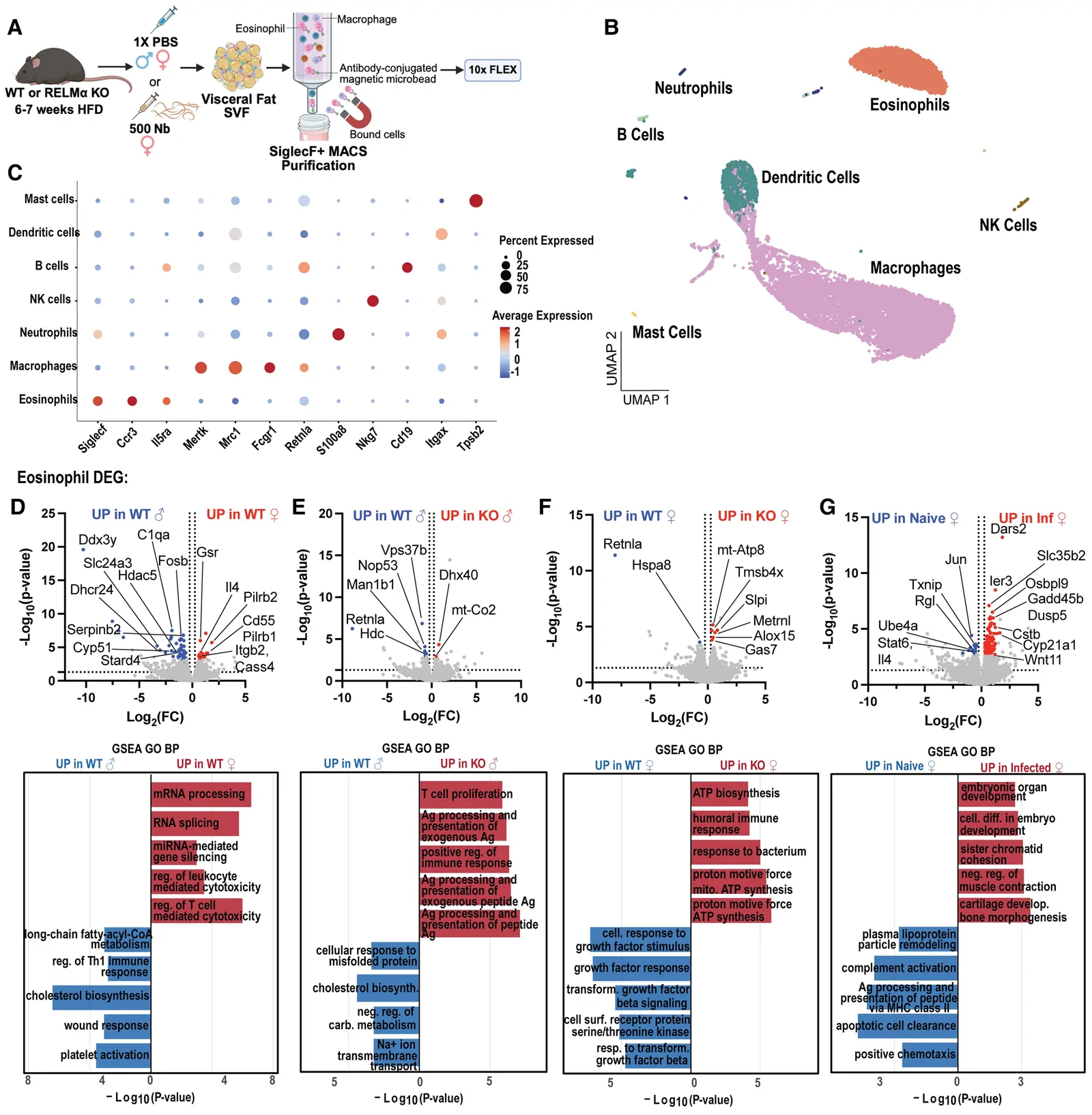
scRNA-seq of adipose eosinophils in diet-induced obesity. A) Experimental design. B) Aggregated UMAP identifying seven immune cell types based on canonical gene signatures. C) Dot plot showing expression of canonical markers across immune cell types; dot size represents percent of cells expressing the gene, color represents average expression level. D–G) Volcano plot for eosinophil DEGs based on sex (D), RELMα, genotype (E, F), and helminth infection (G). Red points indicate upregulated genes (Log2FC ≥ 0.25, *P*_adj_ <0.05), blue points indicate downregulated genes (Log2FC ≤ 0.25, *P*_adj_ < 0.05), and gene ontology biological process (GO BP) pathways for each DEG comparison.

Following quality control single-cell processing (Figs. S3 and S4), pseudobulk differential gene expression analysis was conducted to identify significant differentially expressed genes (DEGs) and gene set enrichment pathway (GSEA) analysis across biological replicates (Fig. 1D–G). When comparing eosinophils from male and female mice (Fig. 1D), there were more genes that were significantly upregulated in males with higher fold induction than females (male 35 genes up, 1.19 log2fold average; female 16 genes up, 0.95 log2fold average). GSEA analysis identified pathways related to cholesterol biosynthesis and wound response (*Slc24a3*, *Dhcr24*, *Serpinb2*, *Cyp51*, and *Stard4*) enriched in male eosinophils. Female eosinophils had GSEA enriched pathways involved in transcriptional regulation (RNA splicing and mRNA processing) and genes involved in migration (*Itgb2)* and *Il4*. Eosinophils from RELMα deficient males had downregulated vesicular trafficking and misfolded protein response pathways (*Vps37b*, *Nop53*, and *Man1b1*), but enrichment in pathways for antigen processing and presentation and T cell proliferation (Fig. 1E). Eosinophils from KO females showed upregulated genes and pathways involved in metabolic reprogramming and innate immune defense, with enriched pathways in ATP biosynthesis and response to bacterium (Fig. 1F). Helminth infection led to an upregulation of genes (Infected vs. Naïve; 110 genes up in infected, 0.72 log2fold average; 21 genes up in naive 0.68 log2fold average), with upregulated genes involved in development and tissue remodeling (*Wnt11* and *Cyp21a1*) and mitochondrial and metabolic stress (*Dars2* and *Slc35b2*) (Fig. 1G).

Collectively, these data identify significant differences in eosinophil gene expression patterns that are especially changed with sex and helminth infection. Pathway analysis indicated a sexually dimorphic functional program shift in eosinophils, with males adopting a transcriptomic profile suited for antigen presentation while eosinophils from females having enriched pathways in metabolism and antimicrobial responses. Additionally, helminth infection triggered gene expression programs involved in metabolic adaptation and structural repair.

### Adipose eosinophil clusters occupy distinct positions along pseudotime trajectories shaped by sex, RELMα, and infection

To quantify transcriptional heterogeneity within the eosinophil population, deep clustering analysis was performed and identified six eosinophil clusters named C0–C5, based on their overall frequency across groups, from high to low (Figs. 2A and S5). Differential gene expression analysis identified cluster-specific gene signatures (Table S1) and biological processes (Fig. 2B–D). Bayesian compositional analysis (sccomp; 31) revealed significant sex-specific cluster composition: C0 (log FC = +1.21, FDR = 0.006) and C2 (log FC = +0.94, FDR = 0.012) were significantly enriched in males. C0 expressed genes for MHC class II antigen presentation (*Cd74*, *H2-Eb1*, *H2-Aa*, and *H2-Ab1*), and C2 was enriched for the male-specific RNA helicase (*Ddx3y*) and genes associated with metabolic adaptation and response (*Dgat1, Srebf2,* and *Glud1*), transcription factor (TF) activity/stress response (*Atf3*, *Nr4a1-3*, *Fosb*, and *Fosl2*) and chemotaxis (*Cxcl2*, *Ccrl2 Il1rl1*) (Fig. 2B and C, Table S1). C1 (log FC = −1.15, FDR = 0.004) and C4 (log FC = −0.87, FDR = 0.016) were enriched in females (Fig. 2D), with *Il4* and integrin subunit beta 2 *Itgb2* enriched in C1, while C4 expressed genes that downregulated activation. For clusters with gene signatures that included surface markers (e.g. C0–C2), high-dimensional spectral flow cytometry on adipose tissue eosinophils was performed for MHCII (C0 blue), LFA-1 (CD18^HI^CD11a^Hi^, C1 pink) and IL-4R/CD124 (C2 orange) (Figs. 2E and S6). HFD males had increased CD124^HI^ Eos (C2), while HFD females had increased CD11a^HI^ Eos (C1). For C0, quantification of MHCII mean fluorescent intensity (MFI) indicated significantly increased MHCII in eosinophils from male mice and with RELMα deficiency (Fig. 2F).

**Figure 2:**
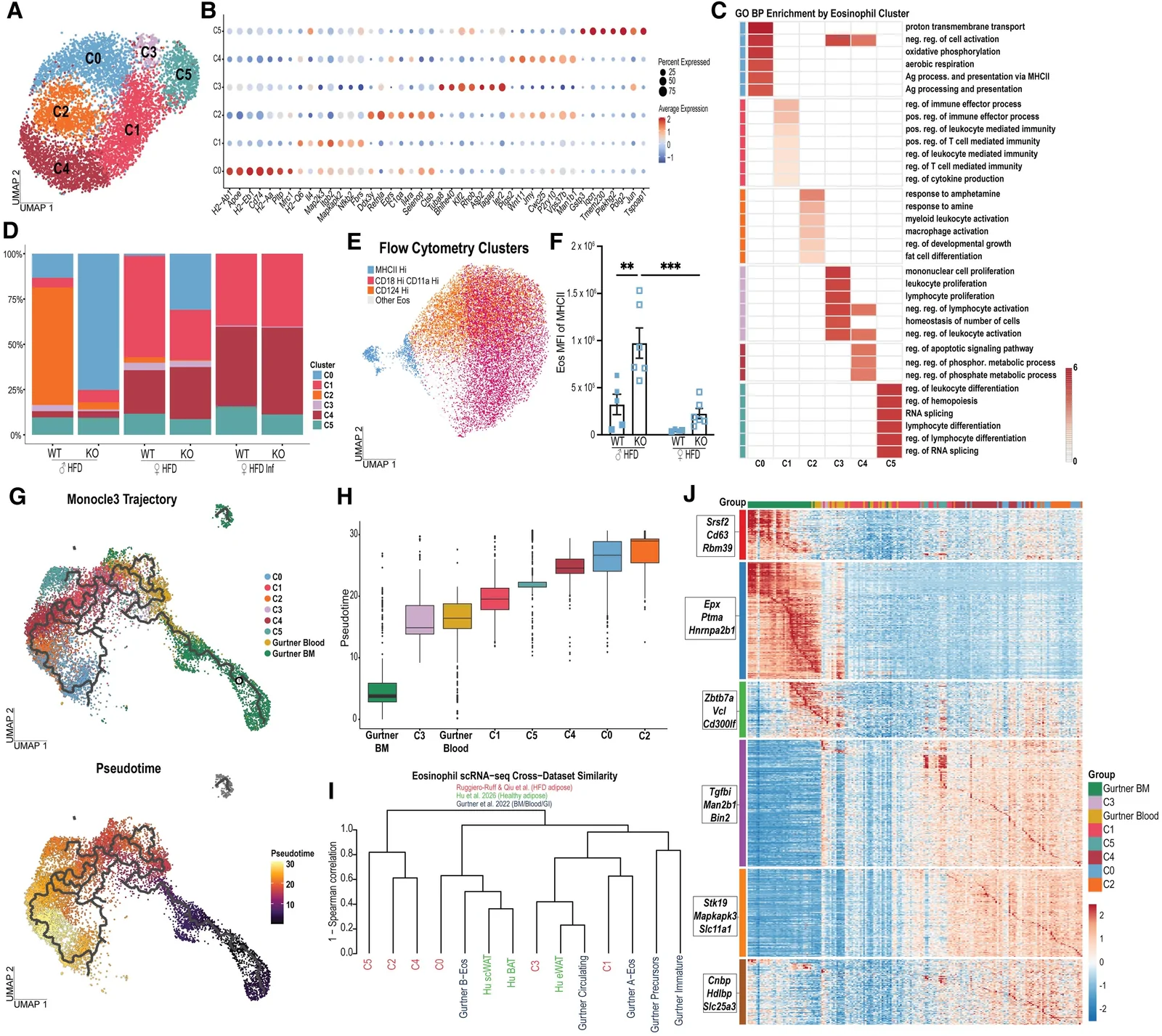
Adipose eosinophil subclusters in diet-induced obesity. A) Aggregated UMAP of 7,461 eosinophils identifying six clusters (C0–C5). B) Dot plot showing scaled expression of the top 6 signature genes for each subcluster. C) Hierarchical clustering of GO BP enrichment heatmap for cluster-specific marker genes based on Benjamini–Hochberg-corrected *P*-values <0.05 and colored by a −log10(*P*_adj_) scale bar. D) Stacked bar graphs of eosinophil subcluster proportions averaged between groups. E) UMAP of high-dimensional spectral flow cytometry analysis of SigecF^+^CCR3^+^Mertk^−^ adipose eosinophil clusters based on cell surface markers. F) MHC2 MFI of adipose eosinophils shown as biological replicates and means ± SEM. Statistical significance was determined by two-way ANOVA with multiple comparisons test (**P < 0.01, ***P < 0.001). G) Monocle 3 trajectory graph of eosinophil clusters integrated with Gurtner et al. bone marrow and blood eosinophils, rooted at the bone marrow population. Colors indicate cluster identity; arrows show inferred trajectory. H) Pseudotime distributions by group and ordered by median pseudotime. Box plots show median, interquartile range (IQR), and whiskers extending to 1.5× IQR. I) Cross-dataset transcriptional similarity dendrogram (Spearman correlation on Harmony-corrected embeddings) comparing adipose eosinophil clusters (C0–C5, red) with Hu et al. healthy adipose depots (eWAT, scWAT, BAT; green) and Gurtner et al. bone marrow and blood populations (blue). J) Gene expression dynamics along pseudotime. Heatmap shows z-scored expression of DEGs ordered by pseudotime, with representative genes labeled per module.

Developmental relationships among eosinophil clusters were determined by Monocle 3 trajectory inference (32) and integration with reference bone marrow and blood eosinophils from Gurtner et al. (2) (Fig. 2G–I). Ordering from bone marrow and circulating-like states toward adipose-adapted eosinophil clusters, including migratory (C1), inflammatory (C0), stress response (C2), infection-associated (C4), and metabolically adapted (C5) programs was observed. Notably, C3 exhibited the lowest pseudotime values among adipose clusters and overlapped with blood eosinophils (Fig. 2H), consistent with its proposed role as a tissue-resident population retaining circulating-like transcriptional features. Cross-dataset similarity analysis (Figs. 2I and S7) reinforced these distinctions: C3 clustered most closely with healthy eWAT eosinophils from Hu et al. (33) and circulating blood eosinophils from Gurtner et al. (2), whereas C0, C2, C4, and C5 formed a distinct adipose-specific branch separated from BM, blood, and intestinal eosinophils. C1 eosinophils clustered closely with Gurtner et al. active eosinophils from the colon, suggesting their role in immune modulation and host defense. Lastly, we determined gene expression dynamics along pseudotime, which delineated sequential maturation stages (Fig. 2J). Bone marrow eosinophils, which were found in early pseudotime, were enriched for genes involved in RNA splicing (*Srsf2* and *Rbm39*) and *Cd63*, a marker for exosome biogenesis that has been implicated in eosinophil piecemeal degranulation (34). Additionally, bone marrow eosinophils upregulated *Epx*, a primary granule protein found in mature, activated eosinophils. Mid-stage pseudotime was marked by upregulation of inflammatory mediators (*Zbtb7a* and *Cd300lf*), cell adhesion and migration genes (*Vcl*, *Tgfbi*, and *Bin2*), and late stage pseudotime gene expression profiles featured chromatin remodeling and DNA repair (*Mapkapk3* and *Stk19*), cholesterol regulation (*Hdlbp*), and mitochondrial function and phagolysosome formation (*Slc25a3* and *Slc11a1*).

Taken together, our results identify heterogeneity in adipose eosinophils that is correlated with the tissue environment and attribute functions of distinct clusters based on their specific transcriptional states. The trajectory analysis positions C3 as a tissue-resident cluster with circulating-like features that may support type 2 immunity and metabolic homeostasis (16), while C0, C2, C4, and C5 represent condition-specific effector states that expand under HFD.

### Eosinophil–macrophage regulatory networks are defined by cluster specialization and chemokine ligand–receptor pairs

Bidirectional crosstalk between eosinophils (Eos) and macrophages (Macs) is known to regulate adipose tissue inflammation and metabolic homeostasis, and our previous work specifically identified a RELMα–Mac–Eos axis as a key driver of female protection in diet-induced obesity (16, 35, 36). CellChat analysis (37) was used to resolve the communication architecture of this axis at higher resolution. Adipose macrophages were classified using a module score approach based on core signature genes for M1-like, M2 surface, and M2 functional subsets (Fig. S8), and their interactions with eosinophils were quantified (Fig. 3). Chemokine ligand (CCL), fibronectin (FN1), amyloid precursor protein (APP), CD45, and thrombospondin (THBS) were identified as main interaction pathways between Mac subsets and Eos (Fig. 3A). Strength and directionality (outgoing vs. incoming) of these signaling pathways across experimental groups was determined with WT female HFD as the protected baseline phenotype (12) (Fig. 3B). The FN1 pathway emerged as a key signaling axis, with elevated outgoing and incoming FN1 signals from M1-like Macs in WT males compared to WT females. Macrophages, particularly the M1 subset and M2 functional subsets in RELMα-deficient mice exhibited increased outgoing signals through the APP pathway, which has been implicated in driving chronic inflammation, mitochondrial dysfunction in adipocytes, and systemic insulin resistance (38, 39). In contrast, eosinophils contributed strong outgoing signals via the THBS pathway, known for its roles in tissue hemostasis, extracellular matrix remodeling, and activation of latent TGF-β (40, 41). CCL signaling had high outgoing and incoming signaling in WT female Eos.

**Figure 3:**
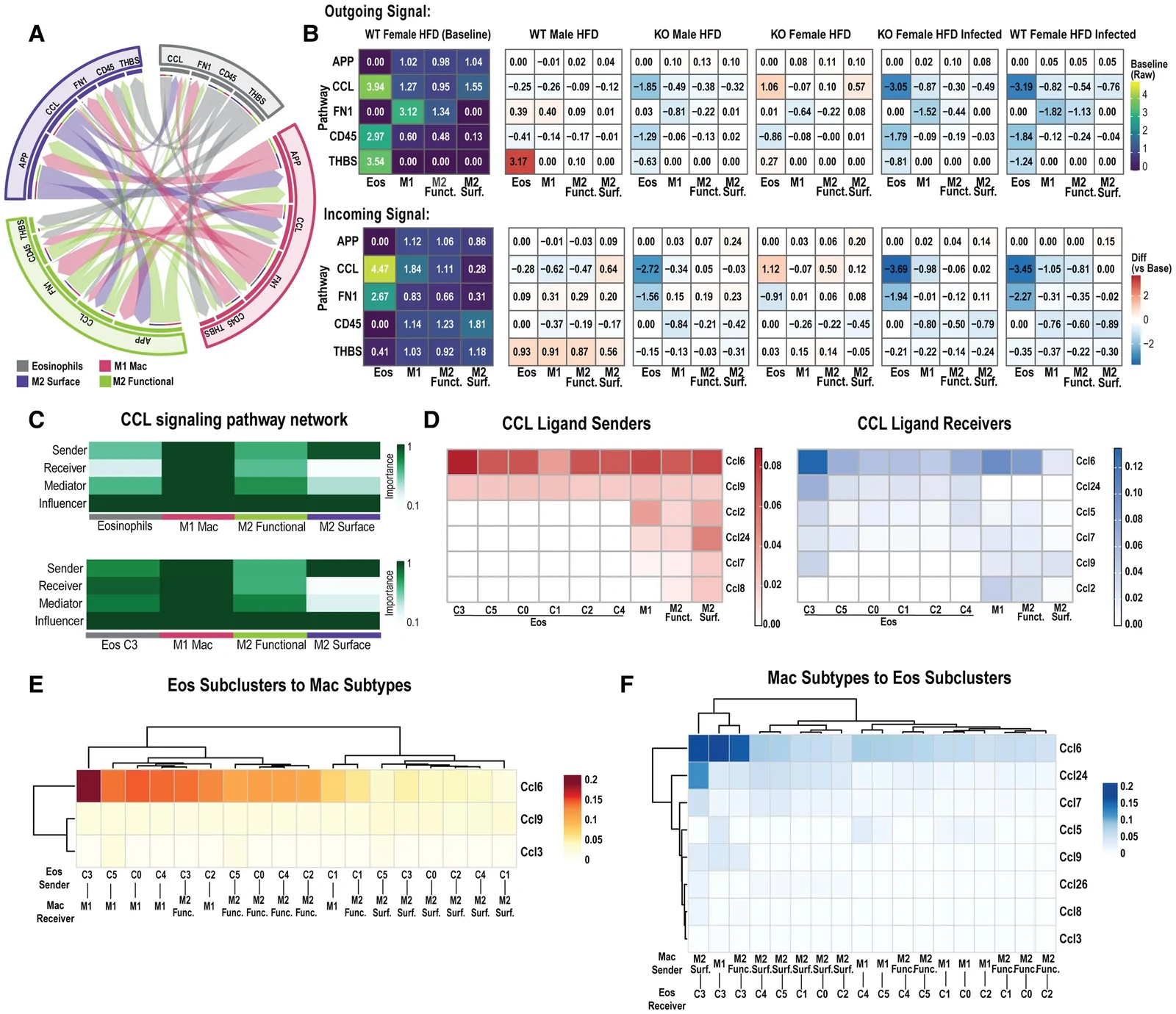
Signaling pathways in adipose eosinophil and macrophage communication across biological groups. A) Chord diagram showing significant interactions (L–R pairs) between eosinophils and macrophage subsets. Arrow direction indicates signaling directionality and thickness indicates signaling strength. B) Heatmaps of outgoing (top) and incoming (bottom) signaling pathway strength across all six experimental conditions (WT female HFD as baseline). Color scale indicates relative signaling strength (red = increased, blue = decreased vs. baseline). C) Heatmap showing the relative importance for each cell type as sender, receiver, mediator, and influencer, based on the computed four network centrality measures of CCL signaling for all eosinophils (top) and C3 cluster (bottom) and macrophage clusters. D) CCL ligand sender (left) and receiver (right) heatmaps showing communication probability between cell types. E, F) CCL ligand–receptor pair strength shown as a heatmap for Eos-to-Mac signaling and reciprocal Mac-to-Eos signaling.

Within the CCL signaling network (Fig. 3C), network centrality analysis (37) identified M1 Macs as the dominant cell type across all signaling roles, M2 Macs as top senders and influencers, and Eos as predominant influencers. Eos cluster C3 was identified as the central cluster in the CCL network—both a key receiver and influencer, and had the highest probability of being a CCL6 sender and receiver (Fig. 3D). Eos C3 primarily signaled to M1 Macs via CCL6, while all Macs signaled back to Eos C3 through CCL6, suggesting a bidirectional CCL6-mediated crosstalk between Eos and Macs (Fig. 3E and F). A second significant interaction involved M2 surface macs communicating with Eos C3 through CCL24 (eotaxin-2), suggesting M2 Macs may recruit and maintain the Eos C3 (Fig. 3F).

Investigation of the CCL–receptor interactions and directionality across biological groups indicated that Mac-to-Eos signaling was enriched for CCL6–CCR3 and CCL24–CCR3 whereas Eos-to-Mac signaling was enriched for CCL6–CCR2 (Fig. 4A and B). M1 and M2 functional Macs exhibited the strongest outgoing CCL6–CCR3 signals to Eos C3 in females compared with males. In the reverse direction, M1 Macs served as the primary receivers of CCL6–CCR2 signals originating predominantly from Eos C3. In WT females, Mac-derived CCL6 is consistent with a candidate role for CCR3-expressing Eos C3 cells to sustain a tissue-resident, immunoregulatory phenotype. This Eos-to-M1 axis was stronger in WT females and decreased in WT males and KO females, suggesting that Eos C3 from WT females may signal to M1 Macs via CCL6–CCR2 circuit to reduce the effects of adipose inflammation from M1 Macs. To determine the spatial distribution of the CCL6–CCR2–CCR3 signaling axis, in situ hybridization was performed on visceral adipose tissue sections, followed by confocal z-stacks of the crown-like structures (CSL) (Fig. 4C and D). WT females exhibited significantly higher frequency of CCR3^+^ cells than WT males (Fig. 4E), which was diminished with RELMα deficiency. Conversely, RELMα-deficiency increased CCL6^+^ cell frequency, particularly in females (Fig. 4F). G-cross function analyses to measure physical proximity between signaling molecules indicated that spatial clustering between CCR3 and CCR2 puncta—a proxy for Eos–Mac crosstalk—was significantly elevated in WT females compared to males but was lost with RELMα deficiency (Fig. 4G). CCL6 to CCR2 proximity demonstrated a significant sex-dependent interaction, with females having higher spatial correlation than males (Fig. 4H), while spatial correlation between CCL6 and CCR3 was unchanged (Fig. 4I).

**Figure 4:**
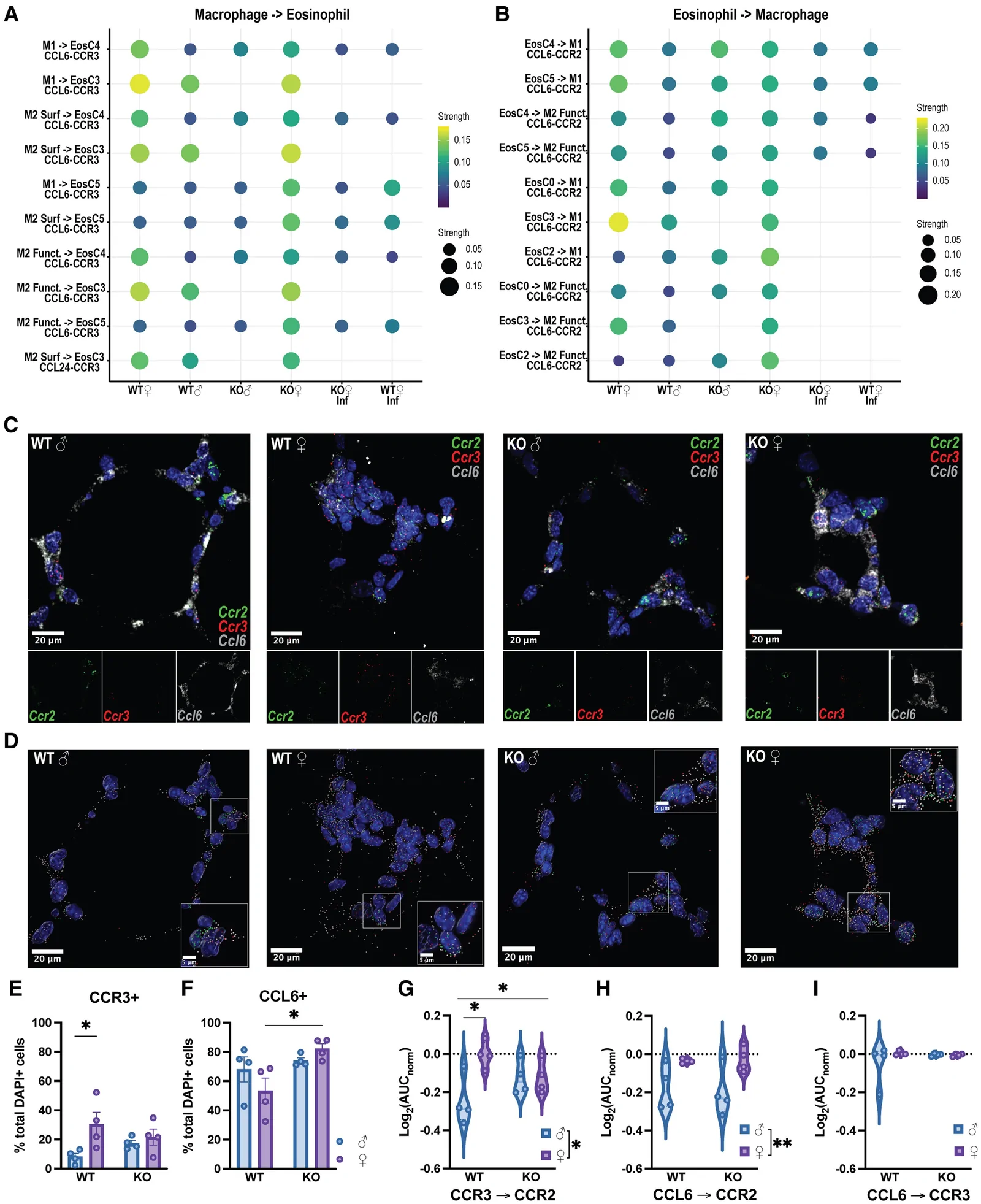
CCL–receptor pairs mediate macrophage-eosinophil networks in the adipose tissue. A, B) Top 10 CCL-mediated signaling interactions between eosinophils and macrophages across experimental groups. Dot plots showing the top 10 strongest CCL (chemokine ligand) signaling interactions (A) from Eos to Mac clusters (A) or from Mac-to-Eos clusters (B). C) RNAscope in situ hybridization confocal microscopy images of representative crown-like structure z-stack projections in visceral adipose tissue stained with *Ccr2* (green), *Ccr3* (red), and *Ccl6* (gray) probes. D) 3D reconstruction of representative confocal images presented in (C) using Imaris. White boxes highlight a zoomed in representative area of the crown-like structure with a *Ccr2–Ccr3–Ccl6* chemokine gradient. E, F) Bar chart comparisons of CCR3- and CCL6-positive cells between experimental groups, shown as a percentage of the total DAPI-positive cells. Positive gene expression was determined by puncta count minimum defined by distribution thresholds. G–I) Violin plots displaying the results of G-cross analysis. Log_2_(AUC_norm_) represents the incidence of high-expressing CCR2 (G, H) and CCR3 (I) cells within proximity to high-expressing CCR3 (G) and CCL6 (H, I) cells as compared to random distribution of gene expression. Statistical significance was determined by two-way analysis of variance (ANOVA) with multiple comparisons test (*P < 0.05, **P < 0.01).

Collectively, these results identify a combinatorial signaling network between adipose eosinophils and macrophages in obesity mediated by shared CCL/CCR ligand–receptor pairs. CellChat analyses indicate that M1, M2 surface, and M2 functional macrophage subsets serve as the major communication hubs, with M1 Macs exhibiting the strongest overall predicted interaction strength. CCL signaling is broadly distributed across Eos clusters, especially Eos C3, where CCL6 shows the highest interaction probability from multiple Eos clusters toward M1 and M2 functional Macs, while CCR3-mediated signaling is enriched toward M2 surface Macs. Lastly, spatial analysis confirmed that RELMα is a critical determinant of the spatial and cellular architecture within the obese adipose niche, potentially by orchestrating sex-dependent chemokine gradients and Eos–Mac proximity.

### Distinct transcriptional regulons define eosinophil clusters in the adipose tissue

We employed per-cluster single-cell regulatory network inference and clustering (SCENIC) analysis (42) to infer gene regulatory networks (GRNs) and TF activity at single-cell resolution. The pipeline was run independently on each eosinophil cluster to enhance detection of cluster-specific regulatory signals while minimizing signals shared across broader cell types. This approach identified distinct regulons with cluster-enriched activity patterns that differed markedly from those in macrophage clusters (Fig. 5A). Eosinophil cluster-enriched regulons showed high cluster specificity and minimal activity in macrophage clusters, confirming that these regulatory programs are eosinophil-intrinsic rather than shared across myeloid populations. Each eosinophil cluster was characterized by a unique set of regulons: C0 by *Zfp959*, *Grhl1*, and *Pole4*; C1 by *Hoxc10*, *Hoxc6*, and *Zfp174*; C2 by *Stat4*, *Zfp729b*, and *Mxd1*; C3 by *Ebf2*, *Cebpg*, *Rel*, and *Nfkb1*; C4 by *Epas1*, *Zfp710*, and *Rcor1*; and C5 by *Hoxa3*, *Nr4a3*, and *Hsf4*. Regulon activity distributions across conditions confirmed sex- and genotype-dependent variation, with per-sample biological replicates showing concordant AUCell scores (Fig. S9).

**Figure 5:**
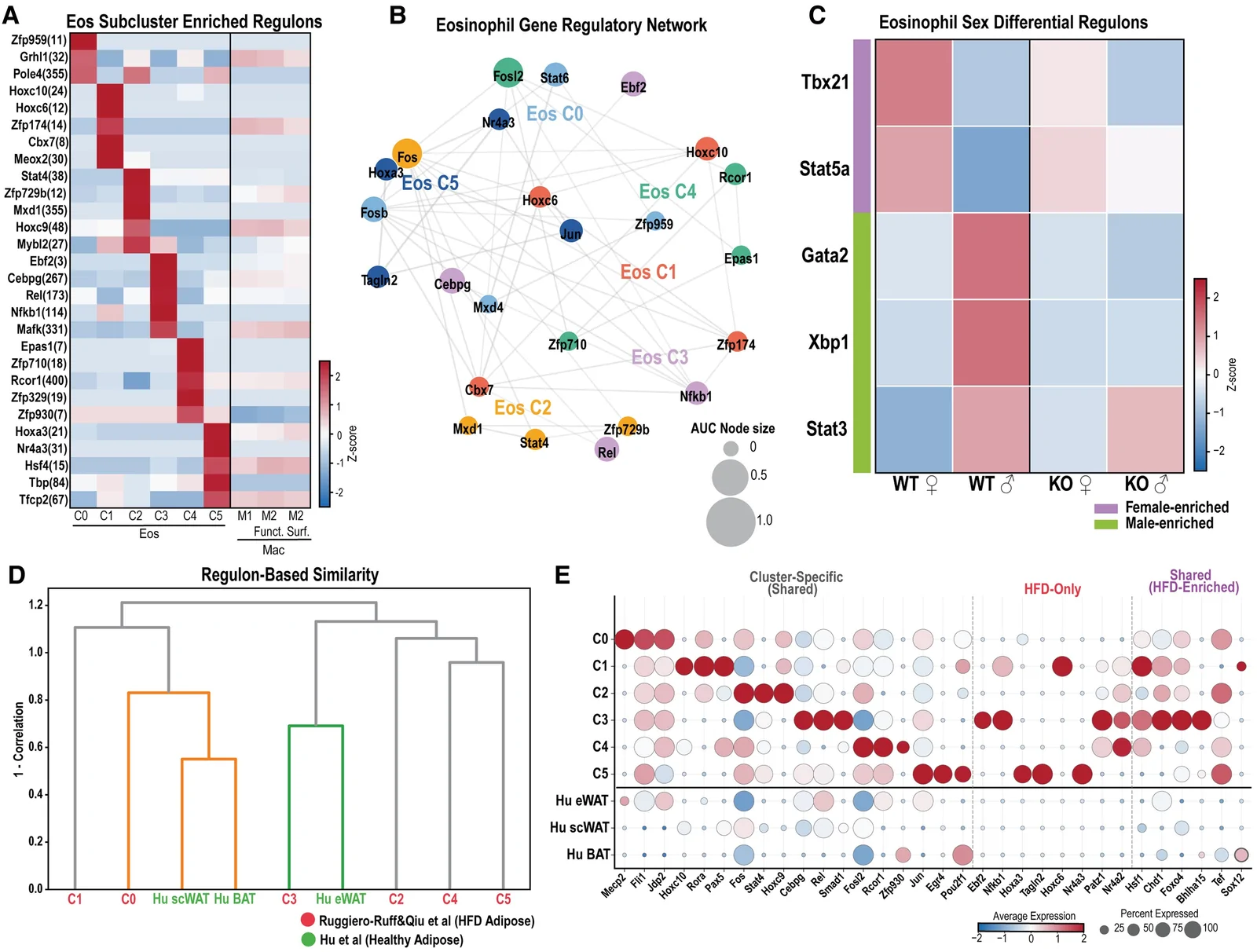
SCENIC analysis identifies adipose eosinophil cluster-specific, sex-differential, and HFD-enriched GRNs. A) Heatmap of cluster-enriched regulon activity (z-scored AUCell scores) across eosinophil clusters (C0–C5) and macrophage clusters (M1, M2 functional, M2 surface). Regulons were identified by per-cluster pySCENIC (GRNBoost2 → cisTarget → AUCell) and selected by cluster specificity. Numbers in parentheses indicate the number of target genes in each regulon. B) Eosinophil GRN map. Nodes represent TF regulons, colored by dominant eosinophil cluster. Node size is proportional to mean AUCell score in the dominant cluster. Edges represent coactivity correlations (Pearson *r* ≥ 0.4) across subsampled cells. Cluster labels indicate spatial groupings. C) Sex-differential regulon activity in adipose eosinophils. Heatmap shows Z-scored mean AUCell regulon activity for five transcription factors with validated sex-dependent activity. Regulons were identified by per-cluster pySCENIC and compared between WT female and WT male using Mann–Whitney *U* test (*P* < 0.05). Only TFs whose SCENIC-identified target-gene expression confirmed the same sex-biased direction (Wilcoxon, *P* < 0.05) are shown. D) Regulon-based cross-dataset similarity dendrogram comparing eosinophil clusters from HFD adipose (red) with healthy adipose depot eosinophils from Hu et al. Hierarchical clustering was performed on Z-scored mean AUCell profiles of shared regulons using correlation distance and average linkage. C0/C1 cluster with Hu scWAT/BAT, and C3 clusters with Hu eWAT, while C2/C4/C5 form a separate HFD-specific branch. E) Dot plot (AUCell > 0.01) of regulon activity across eosinophil clusters (C0–C5) and Hu healthy adipose depots (eWAT, scWAT, and BAT). Regulons are grouped into three categories: cluster-specific shared regulons (detected in both datasets), HFD-only regulons (detected exclusively in HFD adipose eosinophils), and shared HFD-enriched regulons (detected in both datasets but with higher activity under HFD).

Notably, the tissue-resident Eos C3 cluster was enriched for NF-κB family members (*Rel* and *Nfkb1*), consistent with prosurvival and immune-regulatory signaling that supports eosinophil retention and type 2 cytokine production in adipose tissue. In contrast, the male-enriched inflammatory clusters C0 and C2 exhibited AP-1 family regulons (*Fosb* in C0 and *Fos* in C2), aligning with stress-responsive and proinflammatory activation observed under HFD conditions. GRN analysis further inferred interactions among regulons across Eos clusters (Fig. 5B). *Rel* and *Nfkb1* (C3-enriched) occupied central hub positions, bridging regulons from the migratory C1 cluster (*Hoxc10* and *Hoxc6*) and the infection-enriched C4 cluster (*Epas1* and *Zfp710*). The AP-1 family showed modular distribution, *Fosb* (C0), *Fos* (C2), *Fosl2* (C4), and *Jun* (C5), suggesting cluster-specific utilization of this stress-responsive TF family within the obese adipose microenvironment. This interconnected yet modular architecture indicates that terminal tissue adaptation in Eos is achieved through distinct regulatory modules, enabling phenotypic plasticity while preserving core eosinophil lineage identity.

We evaluated whether eosinophil regulatory programs are influenced by biological sex across all subclusters (Fig. 5C). *Tbx21* and *Stat5a* were enriched in females, suggesting that female Eos are more primed to be immunoregulatory and increased survival due to higher sensitivity to IL-5 signaling. In males, *Gata2*, *Xbp1*, and *Stat3* were enriched, suggesting that male Eos retain progenitor-like transcriptional programs and are more proinflammatory and secretory in their function. RELMα deficiency reduced the sex-specific differences, supporting a role for RELMα in stabilizing sex-dependent transcriptional programs in adipose eosinophils.

We next determined if HFD adipose eosinophil transcriptional programs were unique to the HFD tissue microenvironment and if there were fat depot-specific regulatory programs. We integrated healthy adipose tissue eosinophils from Hu et al. (33) from visceral, subcutaneous, and brown adipose tissue and performed SCENIC analysis to determine regulon similarity across datasets (Fig. 5D and E). Dendrogram clustering of regulons revealed our C3 cluster was most like healthy eWAT Eos, while C2, C4, and C5 clustered separately, suggesting that these Eos have transcriptional regulatory programs induced by the obese adipose microenvironment (Fig. 5D). Regulon expression across Eos subclusters and Hu et al. healthy adipose Eos was evaluated and identified obesity-enriched regulons, including *Nr4a3*, *Ebf2* and *Foxo4* (Fig. 5E). The induction of *Nr4a3* (Nor-1) in C5 suggests an activated effector state, as this TF has been shown to be a critical regulator of inflammatory responses under metabolic stress (43). Additionally, the enrichment of *Ebf2*, a master regulator of thermogenic “beiging,” in C3, implies that Eos C3 in obesity may attempt to modulate adipocyte metabolism to counteract obesity-mediated metabolic dysfunction (44). This adaptive regulatory program is complemented by enrichment of *Foxo4*, which likely supports eosinophil persistence by managing oxidative stress within the proinflammatory adipose microenvironment (45). Collectively these data identify differential regulon activity and candidate regulatory programs that contribute to eosinophil transcriptional programs within the adipose tissue microenvironment in obesity. Future functional studies will be necessary to determine whether these regulatory programs dictate eosinophil function in obesity.

## Discussion

This study provides a single-cell resolution of the RELMα–macrophage–eosinophil axis, which we previously identified as a critical mediator of female protection from diet-induced obesity (12). Beyond our earlier findings that RELMα and eosinophils are necessary for inflammatory rescue; we now characterize six adipose eosinophil transcriptional clusters and the unique communication pathways that define their interaction with macrophages within the adipose tissue microenvironment.

Our analysis identifies adipose eosinophils as transcriptionally heterogeneous, resolving them into transcriptional signatures that are dynamically remodeled by biological sex, helminth infection, and RELMα status. This remodeling could contribute to the changes in the eosinophil–macrophage communication network. Whether these eosinophil clusters autonomously drive macrophage polarization or if RELMα functions as a homeostatic stabilizer within this communication network remains a pivotal question for future studies. Our data support a model in which the pairing of specific macrophage and eosinophil transcriptional clusters constitute a recurrent regulatory motif that maintains type 2-polarized immune environments and is likely governed by tissue-resident cues. While the pathways and outcomes vary, this reciprocal interplay between macrophages and eosinophils may be a conserved feature across multiple tissues, including the skin (35), liver (46), lungs (47), heart (48), and skeletal muscle (49). Specifically, in the adipose tissue, our analyses find CCL6/CCR2–CCR3-, FN1-, THBS-, and APP-associated pathways as candidate components of this network.

Our study provides a critical paradigm shift by uniquely investigating how the interaction between biological sex and metabolic stress rewires the eosinophil transcriptomic landscape within visceral adipose tissue. Rather than viewing tissue eosinophils as static, homeostatic bystanders, our approach reveals that their transcriptional signatures are actively molded by the combination of sex and the obese microenvironment (7). Cluster C2 serves as a striking, concrete example of this sex-by-environment reprogramming; rather than a baseline population merely expressing Y-chromosome markers like Ddx3y, C2 represents a profound gene expression signature and is characterized by the coordinated upregulation of master nuclear regulators (*Cebpb*, *Atf3*, and *Nr4a1-3*), lipid-processing metabolic machinery (*Dgat1*), and environmental sensing receptors (*Il1rl1* and *Csf2ra*). By showing that the intersection of male sex and dietary stress drives such a deeply specialized, tissue-resident profile, these findings underscore that understanding metabolic immunity in eosinophils requires evaluating biological sex as a fundamental variable (50).

Through CellChat and spatial analysis, we identify CCL6 as a previously unappreciated mediator of the immune adipose microenvironment, specifically coordinating macrophage–eosinophil crosstalk within CSL. While the CCL2–CCR2 axis is the canonical driver of macrophage infiltration in obesity, our data suggest a more complex, sex-dependent role for CCL6 in maintaining macrophage–eosinophil crosstalk. Previous literature has characterized CCL6 in mouse models of allergic airway inflammation and metastasis, where it is primarily secreted by eosinophils to promote myeloid recruitment and tissue remodeling (51, 52). Our observation that RELMα-deficiency leads to a significant upregulation of CCL6^+^ cells, particularly in females, suggests that RELMα normally acts as a molecular brake on CCL6 expression in the adipose niche. Further, the reduction of CCR3–CCR2 spatial proximity in RELMα-deficient mice highlights a potential dysregulation of eosinophil recruitment to macrophage-rich CSLs. Given that CCL6 can promote the M2-like alternative activation of macrophages and inhibit autophagy in other tissues (53), its upregulation in RELMα-deficient mice, coupled with the loss of specific receptor clustering, suggests dysregulation in functional communication. This is consistent with a model where RELMα may be required for eosinophils to effectively modulate macrophage-driven inflammation within the adipose tissue.

Proper function of the macrophage–eosinophil axis may depend on tissue-specific molecular “stabilizers.” Taken together with our prior functional study (12), our results support a working model in which RELMα serves as a candidate stabilizer in murine adipose tissue. Higher RELMα abundance is associated with a more homeostatic adipose immune environment, particularly in females, whereas global RELMα deficiency is associated with inflammatory macrophage remodeling and reduced eosinophil abundance. Future studies are needed to confirm this function for RELMα, nonetheless, the concept of functional “stabilizers” in cell–cell interactions may offer a useful framework for identifying molecules that preserve tissue-specific immune homeostasis in inflammatory disease.

Our SCENIC analysis suggests that adipose tissue eosinophil clusters differ in regulatory programs, including NF-κB-, AP-1-, IL-5/STAT-, and metabolic-stress-associated regulons in sex, RELMα and obesity-induced inflammation dependent manner. The enrichment of *Ebf2* and *Nr4a3* specifically under HFD conditions implies an adaptive response, where eosinophils may attempt to stabilize the adipose niche or promote thermogenic beiging to counteract metabolic stress. Furthermore, sexual dimorphism of eosinophil regulon activity, such as *Stat5a* in females and *Stat3* in males may explain the observed sex differences in obesity-mediated inflammation and protection.

While our study determined sex, RELMα and infection-dependent transcriptional programs in obese adipose tissue eosinophils, our findings have limitations. Firstly, while we prioritized the use of biological replicates in each group, the number of replicates is limited, and due to limited number of eosinophils from nontransgenic mice coupled with small sample size, more evidence may be needed to confirm these eosinophil transcriptional states as unique clusters. Secondly, batch-effect correction could have increased the rigor of the analysis of eosinophil subcluster comparisons between experimental groups, although all biological replicates were processed at the same time within the same experiment. Future studies involving larger independent cohorts will be required to validate if heterogeneous transcriptional programs in eosinophil subsets exist and are changed in various tissue microenvironments.

In conclusion, our findings have broader implications for our understanding of the role of other TDCs, such as neutrophils, mast cells, platelets, and NK cells in regulating tissue immune status. TDCs offer a distinct evolutionary advantage over reprogrammable immune cells: TDCs predefined transcriptional and proteomic states allow for rapid switching that cells regulated by chromatin remodeling cannot match. However, this comes with a trade-off in flexibility. Rather than broad plasticity, TDCs appear to navigate limited, alternative trajectories of maturation (e.g. homeostatic vs. inflammatory). When the inflammatory trajectories are engaged—or when feedback loop stabilizers such as RELMα fail—the regulatory mechanism collapses, leading to disease. Thus, the heterogeneity identified here in adipose eosinophils may represent a universal mechanism by which TDCs fine-tune tissue immunity, balancing immediate responsiveness with long-term regulation.

## Materials and methods

### Mouse studies

All animal experiments were approved by the Institutional Animal Care and Use Committee at the University of California, Riverside. RELMα KO mice, generated as previously described (12, 54), and C57BL6/J WT controls were bred and maintained under standard 12-h light/dark cycles. At 4–5 weeks of age, mice were randomly assigned to experimental groups and fed a HFD (60% kcal from fat; Research diets) for 6–14 weeks. For single-cell RNA sequencing (scRNA-seq) infection studies, female mice on HFD for 5 weeks were infected subcutaneously with 500 *N. brasiliensis* L3 larvae, as described previously (54, 55), and euthanized 10 days postinfection. To minimize circadian variation, all mice were euthanized between 8:00 AM and 9:00 AM at designated endpoints (10–11 weeks of age for scRNA-seq studies; 16–19 weeks of age for flow cytometry and RNAscope studies).

### Eosinophil enrichment and flow cytometry

Visceral adipose tissue was isolated from PBS-perfused mice, and single-cell suspensions of the stromal vascular fraction (SVF) were obtained with the Adipose Tissue Dissociation kit and gentleMACS Octo Dissociator (Miltenyi Biotec). Eosinophils were enriched via magnetic positive selection using biotinylated antimouse SiglecF antibodies (Biolegend) and antibiotin microbeads (Miltenyi Biotec). High-dimensional spectral flow cytometry was performed on the NovoCyte Opteon (Agilent Technologies). Data analysis, including UMAP visualizations, was conducted using FlowJo v10. Full antibody panels are available in the Supplementary Methods.

### RNAscope in situ hybridization

In situ hybridization was performed on formalin-fixed, paraffin-embedded gonadal adipose tissue sections using the RNAscope Multiplex Fluorescent Reagent Kit v2 (ACD Biosciences). Sections were hybridized with multiplexed probes targeting murine *Ccl6*, *Ccr3*, and *Ccr2*, and labeled with Opal fluorophores (Akoya Biosciences). Z-stack confocal images were acquired on a Zeiss 880 microscope. Spatial relationships and enrichment scores of gene expression between cells were calculated using a modified G-cross analysis as described in the Supplementary Methods (56).

### scRNA-Seq and bioinformatics

Enriched SiglecF + SVF cells were fixed and processed using the 10× Genomics Chromium platform with Fixed RNA Profiling chemistry. Multiplexed libraries were sequenced on an Illumina NovaSeq X Plus platform. Raw sequencing files were processed with Cell Ranger 8.0.1 against the mm10 reference genome, and downstream analyses were performed using Seurat (v5.1.0) in R. Stringent quality control metrics-genes detected per cell, UMI counts, mitochondrial content per sample, DecontX ambient RNA scores, and per-sample gene-UMI relationships-validated consistent data quality across all samples (Figs. S3 and S4). Eosinophil clusters were evaluated for condition-specific compositional changes using Bayesian frameworks (*sccomp*), differential gene expression (*DESeq2*), and pathway enrichment (*clusterProfiler*). Clustering resolution (0.4) was selected based on clustree stability analysis, UMAP visualization at multiple resolutions and silhouette scores (Fig. S5). Intercellular communication networks were mapped via *CellChat*. Trajectory inference, data integration with external tissue datasets, and GRN modeling were conducted using *Monocle 3*, *Harmony*, and *SCENIC*, respectively. Within-study clustering was performed on the unintegrated expression space; Harmony was used only for analyses integrating independently generated external datasets. Computational workflows, filtering criteria, and software parameters are detailed in the Supplementary Methods (57–64).

## Supplementary Material

pgag365_Supplementary_Data

## Data Availability

Sequencing data have been deposited in the Gene Expression Omnibus (GEO) under accession code GSE311941. Analysis code is publicly available on GitHub (https://github.com/xqiu625/adipose-eos-atlas).
