# Supplementary material for: Single-cell heterogeneity of adipose eosinophils is shaped by sex and RELMα and linked to macrophage communication networks in obesity
Source: PNAS Nexus. 2026 Oct 24;5(11):pgag365. doi: 10.1093/pnasnexus/pgag365 (PMC13741244; doi:10.1093/pnasnexus/pgag365)
Supplement: Supplementary file 1 [file pgag365_supplementary_data.zip › PNASNEXUS-PNASNEXUS-2026-00330-TRR-s02.pdf]

Supplementary Methods

Spectral Flow Cytometry Panel and Staining Conditions

For immunophenotyping, 10^6 SVF cells were stained with Zombie Aqua Viability dye (Biolegend) to exclude dead cells. Fc receptors were blocked using anti-mouse CD16/CD32 (Biolegend) combined with Rat IgG (Sigma) for 5 minutes on ice. The following fluorophore-conjugated antibodies (Biolegend unless noted) were incubated with cells according to manufacturer recommendations: CD18 (BV421), CD45 (BV570), MerTK (FITC), CD11a (RB705), CCR3 (PE), SiglecF (PE/Dazzle 594), IL-4Rα (APC), CD45 (BV570), CD11b (APC/Cyanine7) and MHC Class II (Alexa Fluor 700, eBioscience). UMAP visualization was generated using the FlowJo v10 UMAP plugin on concatenated samples (5,000 cells downsampled per biological replicate per group) targeting parameters MerTK, CD11a, IL-4Rα, MHC Class II, CD18, CD45, CCR3, and SiglecF. The explicit gating tree (CD45+ CCR3+ SiglecF+ MerTK-) is presented in Fig. S2.

RNAscope Image Processing and Spatial G-Cross Measurement

Perfusion was executed with 20 mL of 1X PBS. Harvested gonadal adipose tissue was fixed in 10% neutral buffered formalin for 24 hours at room temperature, transferred to 70% ethanol at 4°C, and paraffin-embedded at the UCLA Translational Pathology Core Laboratory. Sections (5 and 10 um) were hybridized with C1-CCL6 (510851), C2-CCR3 (576351-C2), and C3-CCR2 (501681-C3) probes (ACD Biosciences) for 2 hours at 40°C. Development was completed with Opal 520 (FP1487001KT), Opal 570 (FP1488001KT), and Opal 690 (FP1497001KT, Akoya Biosciences) fluorophores. Slides were mounted using VECTASHIELD Vibrance with DAPI (H-1800, Vector Laboratories).

Confocal Z-stack images (40X objective) were transformed into standard deviation Z-projections using Fiji ImageJ (v1.54p). Crown-like structures were rendered in 3D using Imaris 11.0. Nuclei and puncta coordinates were exported for spatial G-cross analysis (56). The spatial enrichment score AUCnorm calculated the average area under the curve (AUC) at r'=25um for each reference nucleus, normalized via permutation tests and averaged across images per animal. Thresholds for positive cellular expression

were calibrated dynamically to match dataset distribution patterns based on baseline ACDbio recommendations.

10X Genomics FLEX Library Construction

For scRNA-seq, 50,000–200,000 MACS-enriched cells per sample were fixed in 1 mL Fixation Buffer for 17 hours at 4°C, then neutralized in 1 mL chilled Quenching Buffer. Cells were incubated with unique mouse Whole Transcriptome Atlas (WTA) probe barcodes (4x4 multiplexing) and hybridized for 17 hours at 42°C. Barcoded samples were pooled equally, washed, and loaded onto a Chromium Next GEM Chip Q for Gel Bead-in-Emulsion (GEM) generation. Libraries were prepared with unique sample indices and sequenced at the UC San Diego IGM Genomics Center.

Bioinformatic Quality Control and Preprocessing

Single-cell RNA sequencing (scRNA-seq) was performed using the 10x Genomics Chromium platform with Fixed RNA Profiling chemistry. Libraries were prepared according to the manufacturer's protocol using probe sets optimized for the mouse transcriptome (Chromium Mouse Transcriptome Probe Set v1.0.1). Raw sequencing data was processed using Cell Ranger 8.0.1 (10x Genomics) with the mm10-2020-A mouse reference genome. Single-cell expression matrices were analyzed using Seurat (v5.1.0) (47) in R (v4.4.2). Ambient RNA contamination was estimated and removed using DecontX (celda package) (57) with a contamination threshold of 0.2, applied independently per sample. Doublets were identified and removed using Scrublet (58) with an expected doublet rate of 6%. Cells were further filtered to retain those with 200–5,000 detected features and ≤10% mitochondrial gene content. Data were log-normalized, and the top 2,000 highly variable features were selected. Principal component analysis was performed with 30 components. After subsetting to SiglecF^{+} eosinophils, the standard Seurat workflow (NormalizeData, FindVariableFeatures with 2,000 highly variable features, ScaleData, RunPCA with 30 components, RunUMAP on the first 20 PCs, FindNeighbors, and FindClusters) was re-run de novo on the eosinophil cluster to capture eosinophil-intrinsic variation.

Cell Type Annotation

Cell type identification was performed using two complementary approaches. First, we conducted differential expression analysis using Seurat (v5.1.0) (59) to identify cluster-specific markers, requiring expression in at least 25% of cells and a minimum log2 fold change of 0.25. The top 10 marker genes per cluster were used for initial cell type annotation. Additionally, we employed Universal Cell Embedding (UCE) (60). Using a 33-layer neural network model and k-nearest neighbor (k=5) classification. For macrophage classification, macrophages within the Siglec-F+ sorted population were further classified into three clusters using module scoring (Seurat AddModuleScore) with curated gene sets: M1-like (pro-inflammatory: *Itgax, Cd80, Tnf, Il6, Ccl2*), M2-like surface marker-defined (*Mrc1, Lyve1, Cd301b, Cd163*), and M2-like functionally defined (*Chil3, Il10, Arg1, Tgfb1*, *Clec10a*). Each macrophage was assigned to the cluster for which it scored highest.

Eosinophil subcluster analysis across groups

Clustering resolution (0.4) was selected based on clustree stability analysis, UMAP visualization at multiple resolutions and silhouette scores (Fig S5). To statistically test changes in eosinophil cluster proportions across experimental conditions while accounting for the compositional nature of single-cell data and sample-level pseudoreplication, we performed Bayesian compositional analysis using sccomp v1.7 (31). For each biological replicate, cell counts per cluster were tabulated from the eosinophil subset (7,461 cells across 16 samples). sccomp fits a Beta-Binomial generalized linear model with a sum-constrained framework that properly handles the compositional constraint (proportions sum to one). Three contrasts were tested: 1. sex effect (WT Female vs WT Male, HFD-only samples, n=3 vs n=3; formula: ~sex), 2. genotype effect (KO vs WT, females HFD-only, n=3 vs n=3; formula: ~genotype) and 3. infection effect (HFDInfected vs HFD, all females, n=4 vs n=6; formula: ~infection). For each contrast, the composition model included the variable of interest as a fixed effect and an intercept-only variability model (~1). Model Fitting was performed using variational inference with 50 pathfinder paths. Significance was determined using false discovery rate (FDR)-corrected posterior probabilities, with FDR < 0.1 considered significant. Log fold changes in composition and 95% credible intervals were extracted for each cluster-condition comparison.

Differential gene expression and Pathway Enrichment Analysis

Differential gene expression analysis was performed using a pseudobulk approach with DESeq2. Single-cell counts were aggregated at the sample level, and differential expression was assessed using a multifactor design to account for genotype, sex, and infection status (61). We analyzed eosinophil populations under various conditions including wild-type (WT) and knockout (KO) genotypes, male and female subjects, and HFD (High-Fat Diet) with and without infection status. Genes were considered differentially expressed based on adjusted p-values and log2 fold change thresholds. Gene set enrichment analysis (GSEA) was performed using clusterProfiler's gseGO() function with Gene Ontology Biological Process terms. Genes were ranked by DESeq2 test statistic, and enrichment was assessed with Benjamini-Hochberg adjusted p-values.

Cell-Cell Communication Analysis

Cell-cell communication analysis was performed using the CellChat R package (v1.6.1) (37) to infer and quantify intercellular signaling networks. For each experimental condition, CellChat objects were generated from single-cell RNA sequencing data, and communication probabilities were computed based on the expression of known ligand-receptor pairs in the CellChatDB database. Five signaling pathways (APP, CCL, FN1, CD45, THBS) were selected for analysis. To compare signaling patterns across conditions, differential signaling strength was computed by subtracting the baseline condition (WT Female) values from each comparison condition.

Integration with External Datasets

To contextualize our adipose eosinophil clusters, we performed two levels of data integration using Harmony (v1.2.1) (62) with default parameters on the top 30 PCA components. First, our HFD adipose eosinophils were integrated with healthy adipose eosinophils from Hu et al. to assess cluster conservation across dietary conditions. Cluster co-localization analysis was performed to quantify the overlap between our subclusters and the Hu reference. Second, a three-study integration was performed combining our dataset (HFD adipose), Hu et al. (healthy adipose) (33) and Gurtner et al. (GSE182001; normal blood and bone marrow eosinophils) (2) to map eosinophil maturation from bone marrow precursors through circulating to tissue-resident states. Data from each dataset were processed using Seurat with standard log-normalization and 2,000 highly variable features prior to integration.

Trajectory Analysis of Eosinophil Differentiation

We performed trajectory inference analysis using Monocle 3 (v1.2.9) (32, 63) to examine developmental progression by integrating our eosinophil dataset with reference eosinophils from Gurtner et al. (blood and bone marrow) with Harmony. Trajectory alignment was performed using align_cds (64). To establish the directionality of the trajectories, root nodes were selected based on the Gurtner et al. normal bone marrow population. For the pseudotime gene expression heatmap (Fig 2J), pseudotime-dependent genes were identified using Monocle 3's graph_test (Moran's I test, q-value < 0.05) on the top 1,000 highly variable genes across pseudotime-ordered cells. The resulting genes were grouped into six co-expression modules by hierarchical clustering of their pseudotime expression dynamics, and the top three genes by variance within each module were labeled as representative markers of each maturation stage.

SCENIC Analysis

We applied SCENIC (42) to infer gene regulatory networks and assess transcription factor (TF) activity at single-cell resolution. Co-expression networks were inferred using GRNBoost2 independently per eosinophil subcluster (C0–C5) to amplify cluster-specific regulatory signals. Modules were refined using cisTarget (NES ≥ 3.0, mm10 motif database v9). Regulons were constructed using the mouse motif database (mm10, v9). Regulon activity was quantified using AUCell, which scores the enrichment of regulon genes within the top 5% of expressed genes per cell. The cell-by-regulon AUC matrix was used for downstream comparative analyses.

Differential Regulon Activity Analysis

To identify regulons with differential activity between eosinophil subclusters and macrophage clusters, we performed comparative analyses using a random subsampling approach adapted from Xie et al. 2020 (17). For each cell population, 200 cells were randomly subsampled and mean AUCell scores calculated. Regulons were classified as "Eos-enriched" if they met the following criteria: 1. maximum AUC score across Eos subclusters ≥ 0.05, and 2. fold change versus mean macrophage AUC ≥ 1.2.To identify regulons with sex-dependent activity, mean AUCell scores were computed per condition (WT female, WT male, KO female, KO male) by averaging across conditions for visualization. Therefore the regulon

heatmaps are descriptive summaries, while per-cell distributions and per-sample means are shown in Fig S9.

Gene Regulatory Network Analysis

Gene regulatory networks were constructed to visualize the relationships among enriched transcription factor regulons identified from the SCENIC analysis. For eosinophils, we selected regulons that passed the enrichment and uniqueness filters and quantified their co activity by calculating pairwise Pearson correlations across single cells. Networks were built using NetworkX with edges drawn between regulon pairs with correlation ≥ 0.4. Network modules were detected using the Louvain community detection algorithm to identify densely connected groups of regulons.

Supplementary Table S1. Eosinophil Subcluster Differentially Expressed Genes

Supplemental Figures

Figure S1. Total Body and Visceral Adipose Tissue Weights with Subsequent Cytospin Analysis of SiglecF-Enriched Cells. Animal body weight **(A)** and visceral adipose tissue weight **(B)** of all groups after 6 weeks on high fat diet. **(C)** Representative cytospin preparations of SiglecF-enriched cells from WT Male HFD, KO Male HFD, WT Female HFD and KO Female HFD stained with Diff-Quik. Percentage of Eosinophils **(D)**, Macrophages **(E)** and Mast cells **(F)** counted in SiglecF-enriched cytospins from visceral adipose tissue of n=3 animals per group. All scale bars = 20uM.

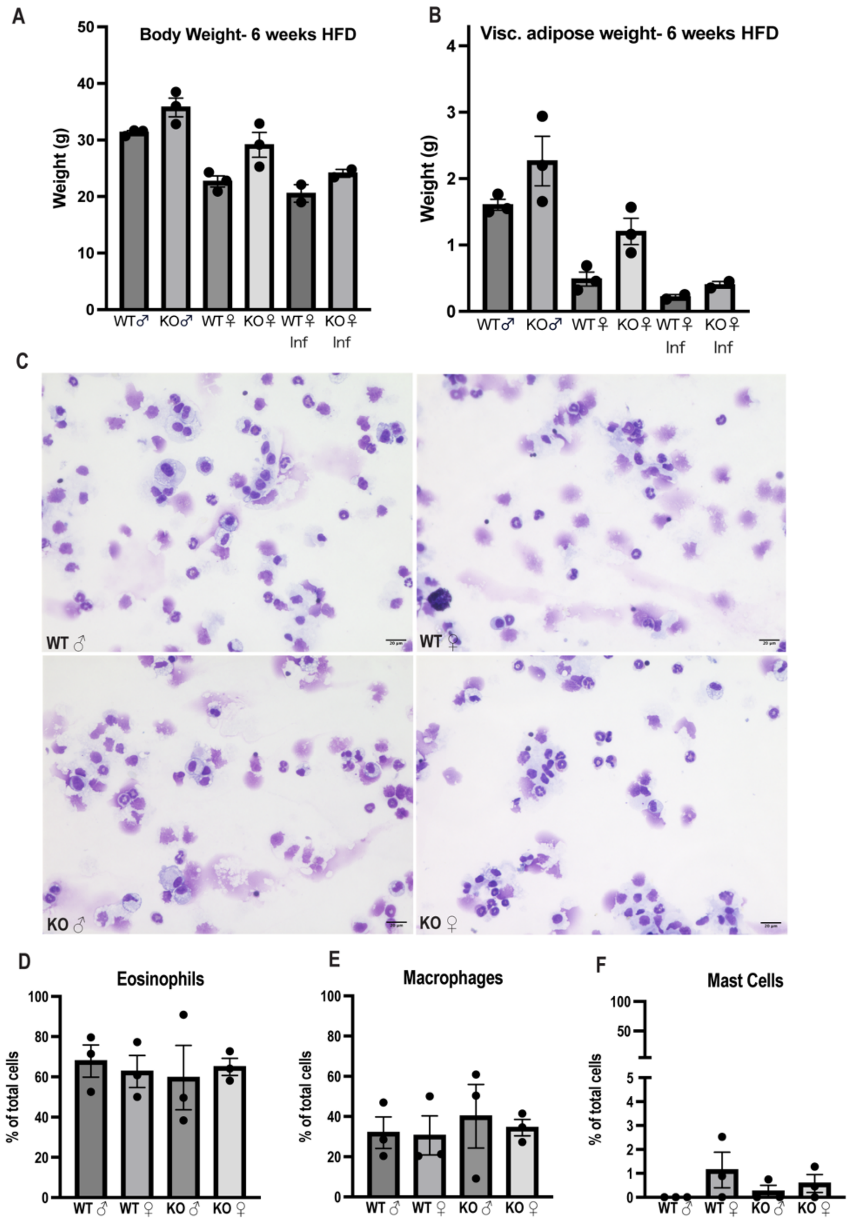

Figure S1

Figure S2. Adipose tissue stromal vascular fraction eosinophil gating strategy and SiglecF enrichment. (A) Representative flow cytometry plots of gating strategy for adipose tissue eosinophils and identification of MHCIIhi, CD124hi and CD18hi CD11ahi eosinophil populations from the visceral adipose tissue stromal vascular fraction. **(B)** Representative flow cytometry plots of SiglecF enriched cells from adipose tissue stromal vascular fraction from full digest (left) and after enrichment (right). Proportions of eosinophils were calculated based on total live cells.

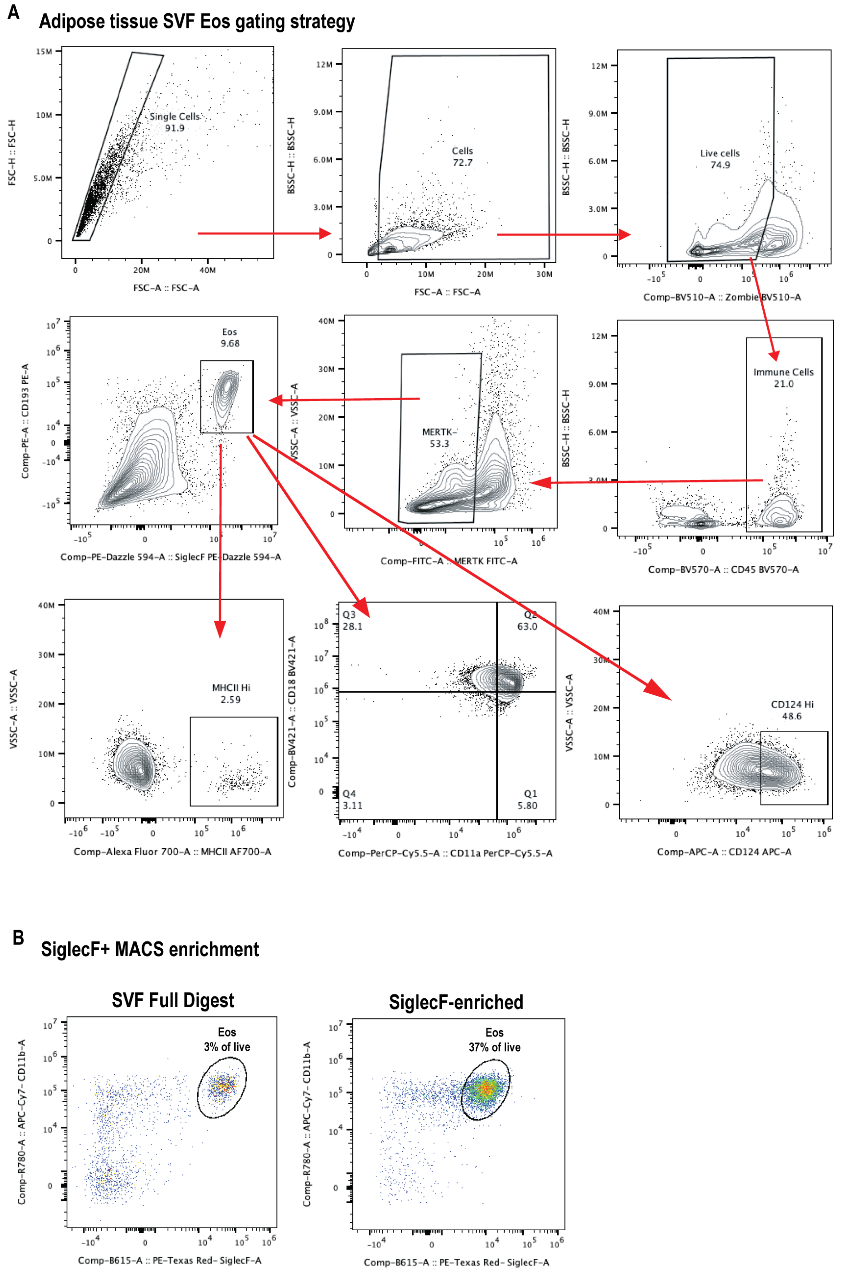

Figure S2

Figure S3. Single-cell RNA sequencing quality control metrics. (A) Violin plots of genes detected per cell (nFeature_RNA) for each of the 16 samples, post-QC filtering (200–5,000 genes, <10% mitochondrial content, Scrublet doublet removal at 6% expected rate). **(B)** Violin plots of UMI counts per cell (nCount_RNA, log10 scale) per sample. **(C)** Violin plots of mitochondrial content per cell per sample; dashed red line indicates the 10% filtering threshold.

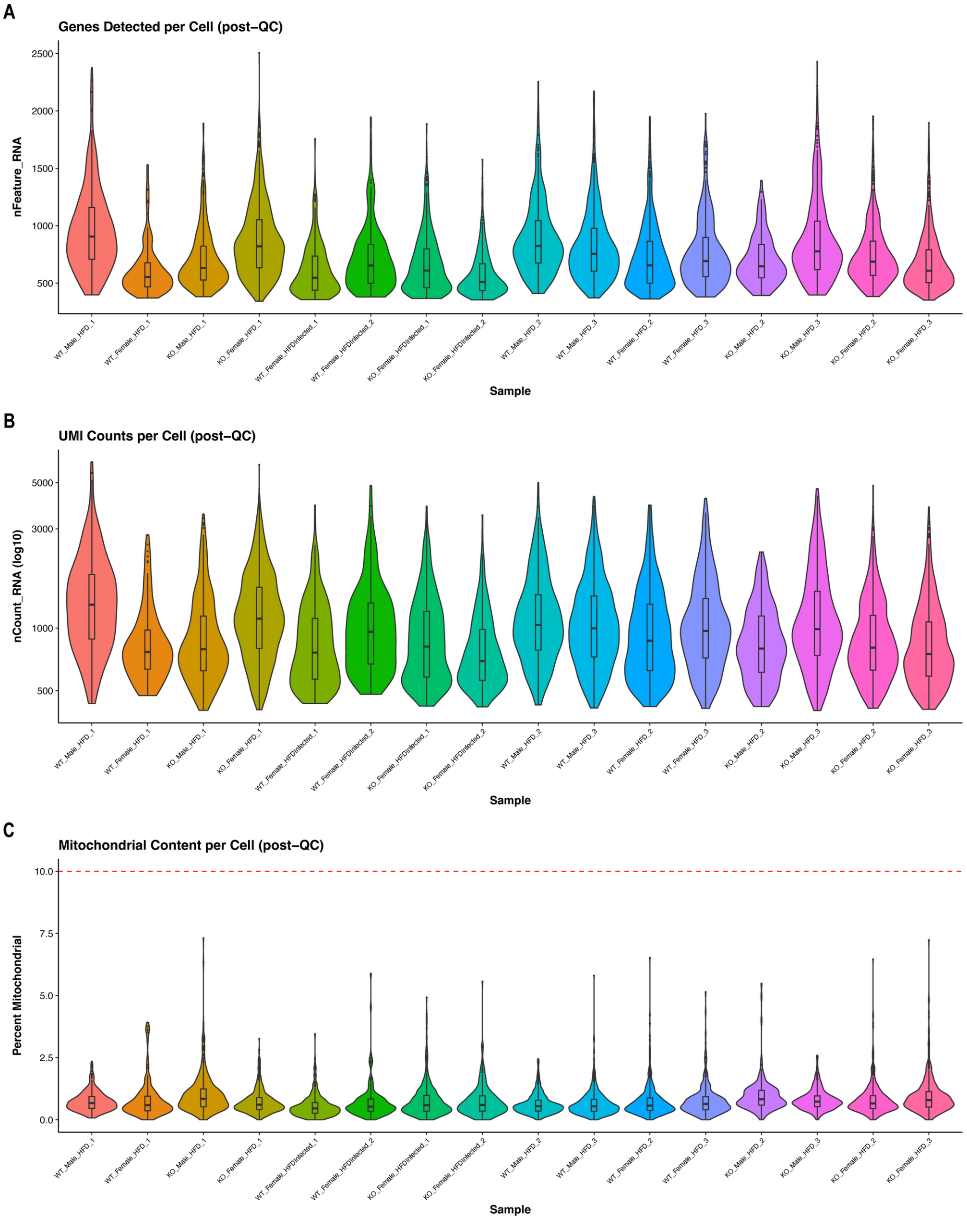

Figure S3

Figure S4. DecontX ambient RNA removal and per-sample data quality. (A) Histograms of DecontX contamination scores for retained eosinophils (C0–C5) per sample. All retained cells have contamination scores below the 0.2 threshold (dashed red line), confirming effective ambient RNA removal. **(B)** Per-sample scatter plots of UMI count (log10) versus gene count, colored by mitochondrial percentage. The absence of discrete high-UMI/high-gene outlier clouds confirm effective doublet removal.

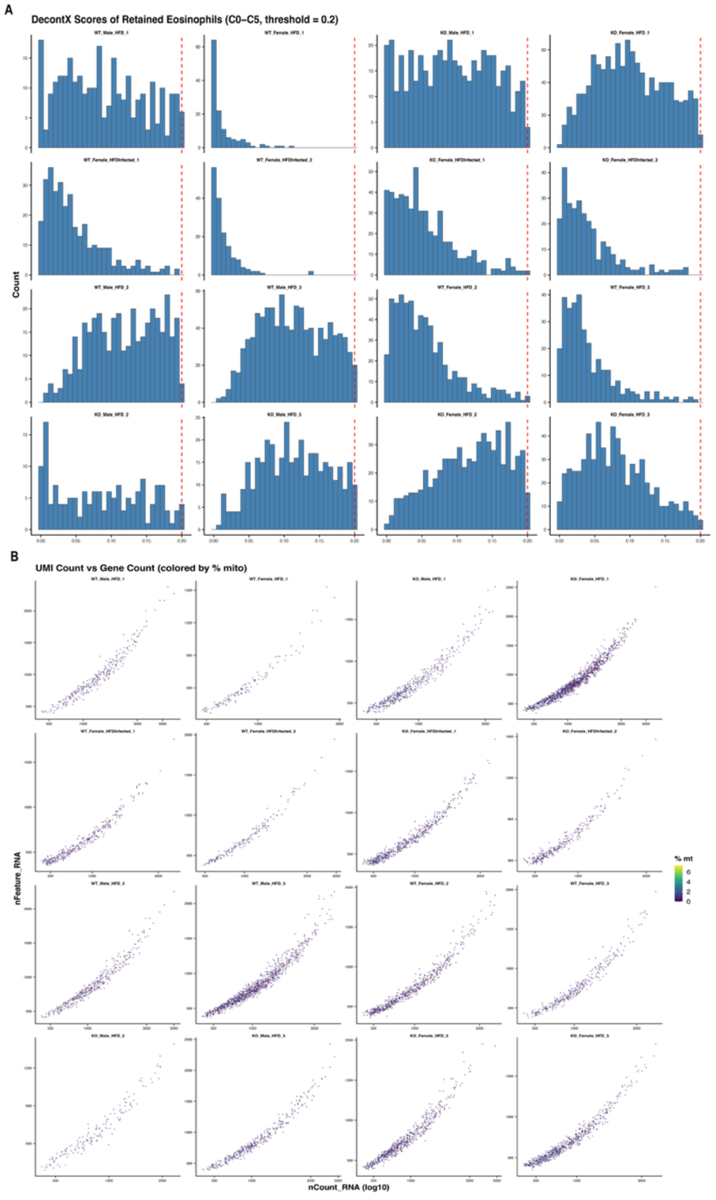

Figure S4

Figure S5. Clustering resolution justification of 10x FLEX dataset. (A) Clustree diagram showing cluster stability across resolutions 0.1–1.5. Node size represents cluster size; arrow opacity represents the proportion of cells from a parent cluster. Clusters C0–C5 emerge stably at resolution 0.3 and persist through 0.6, with fragmentation at higher resolutions. **(B)** UMAP visualizations at resolutions 0.3 (6 clusters), 0.4 (7 clusters, chosen; red border), 0.5 (7 clusters), and 0.8 (8 clusters). **(C)** Left: mean silhouette score versus clustering resolution, showing a plateau between resolutions 0.3–0.5. Right: number of clusters versus resolution. Red diamond indicates chosen resolution (0.4). **(D)** Bayesian compositional analysis of eosinophil cluster proportions across conditions. Forest plot showing log fold changes in cluster composition with 95% credible intervals estimated by sccomp (Beta-Binomial model; 31). Three contrasts are shown: sex effect (Male vs Female, WT HFD-only, n=3 vs n=3), genotype effect (KO vs WT, females HFD-only, n=3 vs n=3), and infection effect (HFDInfected vs HFD, all females, n=4 vs n=6). Red points indicate significant compositional changes (FDR < 0.1); grey points indicate non-significant changes. Horizontal bars represent 95% credible intervals; dashed vertical line indicates no change (log FC = 0) **(E)** Per-sample eosinophil cluster proportions. Stacked bar plot showing the proportion of each eosinophil cluster (C0–C5) within individual biological replicates, grouped by experimental condition (n=2–3 replicates per condition). Each bar represents one sample, demonstrating replicate-level concordance of cluster composition within conditions.

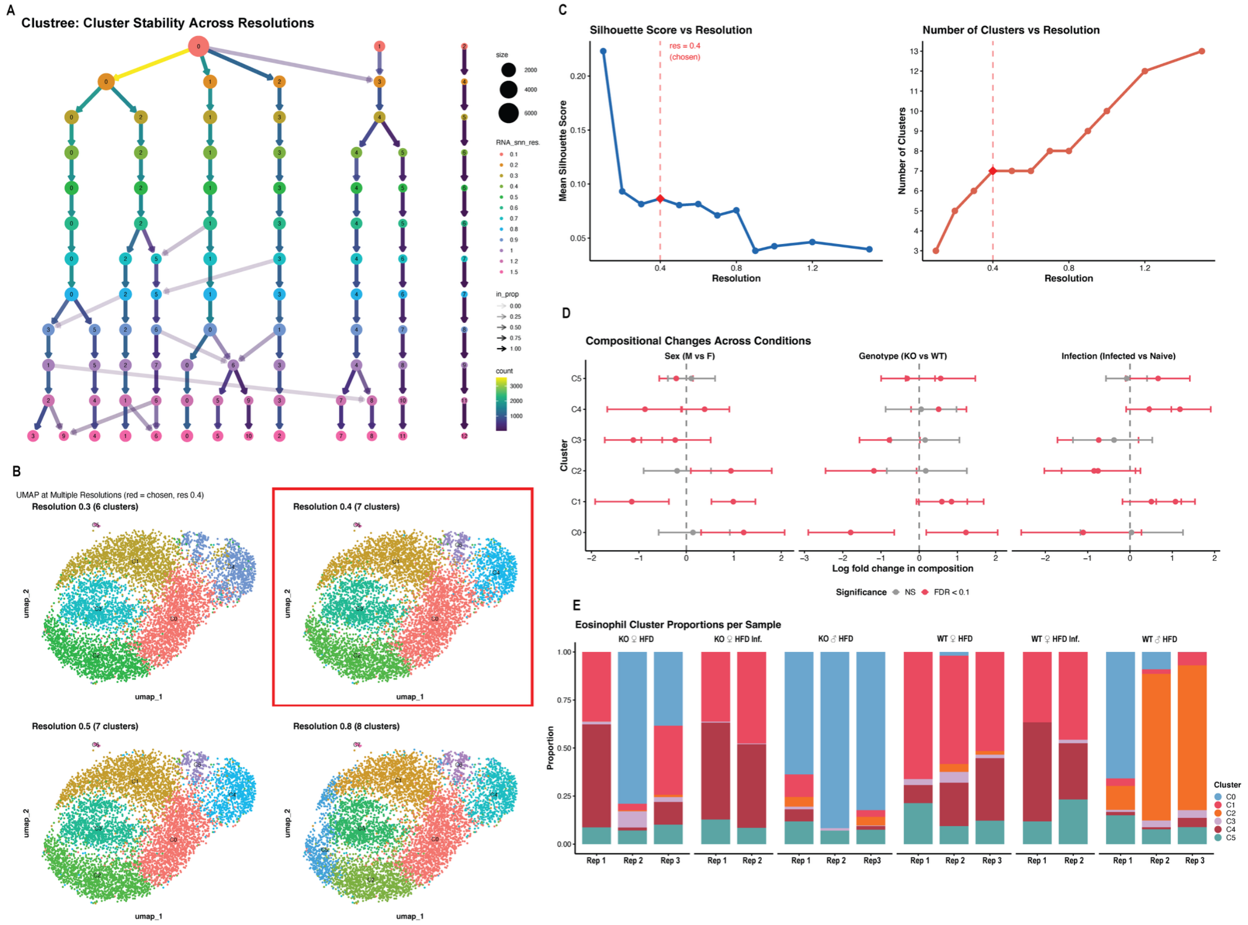
A
Clustree: Cluster Stability Across Resolutions
size
RNA_snn_res.
in_prop
count
B
UMAP at Multiple Resolutions (red = chosen, res 0.4)
Resolution 0.3 (6 clusters)
Resolution 0.4 (7 clusters)
Resolution 0.5 (7 clusters)
Resolution 0.8 (8 clusters)
umap_1
umap_2
C
Silhouette Score vs Resolution
res = 0.4
(chosen)
Mean Silhouette Score
Resolution
Number of Clusters vs Resolution
Number of Clusters
Resolution
D
Compositional Changes Across Conditions
Sex (M vs F)
Genotype (KO vs WT)
Infection (Infected vs Naive)
Cluster
Log fold change in composition
Significance
NS
FDR < 0.1
E
Eosinophil Cluster Proportions per Sample
KO ♀ HFD
KO ♀ HFD Inf.
KO ♂ HFD
WT ♀ HFD
WT ♀ HFD Inf.
WT ♂ HFD
Proportion
Cluster
C0
C1
C2
C3
C4
C5
Rep 1
Rep 2
Rep 3
Rep3

Figure S5

Figure S6. Flow Cytometry UMAP visualization of eosinophil subpopulations. (A) Flow cytometry panel-designed UMAP of normalized concatenated eosinophils aggregated from visceral adipose SVF from WT Male HFD, WT Female HFD, KO Male HFD, KO Female HFD (n=3-5 animals per group). **(B)** UMAP analysis of SVF eosinophils from different groups (n=3-5 animals per group). Clusters are color-coded based on their expression of CD11a (red), CD124 (orange) and MHCII (blue). Data are representative of 2 experiments with 3-5 mice per group.

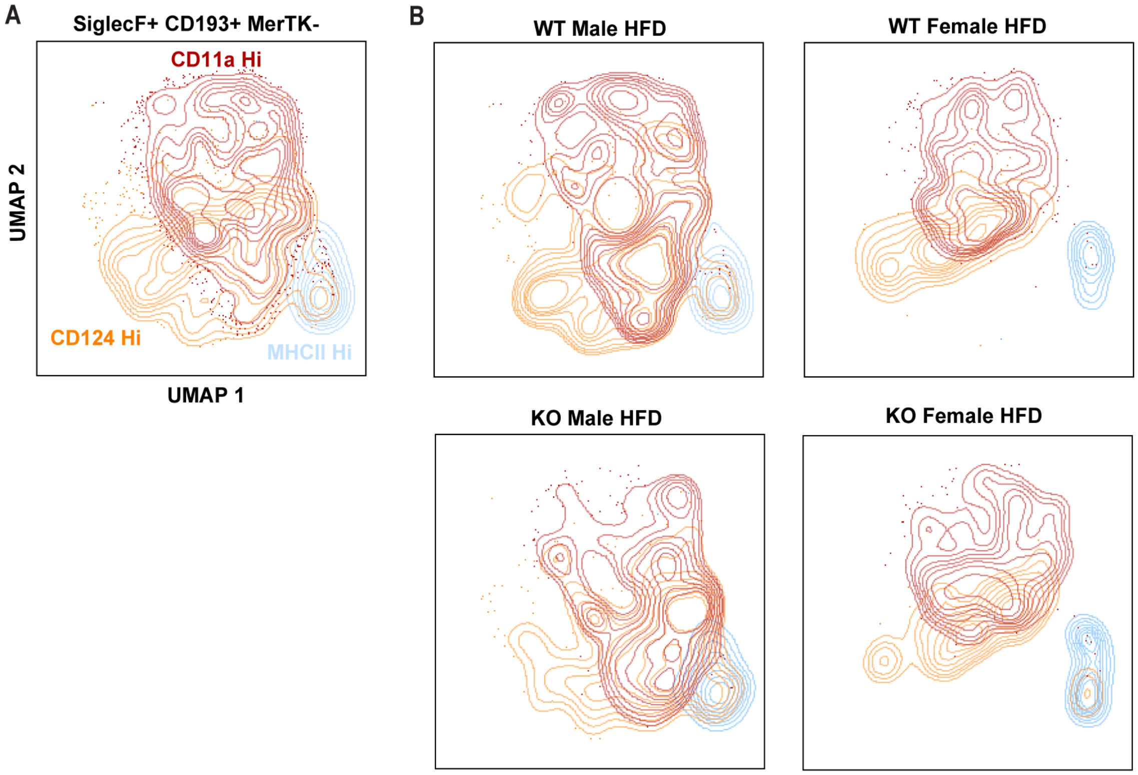

Figure S6

Figure S7. Cross-tissue eosinophil comparison with Gurtner *et al.* intestinal eosinophils. (A) Integrated UMAP of Ruggiero-Ruff and Qiu *et al.* adipose eosinophils (red) with Gurtner *et al.* bone marrow (dark blue), blood (light blue), colon (gold), and small intestine (brown) eosinophils after Harmony integration. **(B)** Feature plots of canonical eosinophil markers (*Siglecf*, *Ccr3*, *Il5ra*, *Prg2*, *Epx*) on the integrated UMAP, confirming eosinophil identity across all tissue sources. **(C)** Spearman correlation heatmap of average Harmony embeddings across all groups. Hierarchical clustering separates adipose-specific clusters (C0, C2, C4, C5) from circulating-like clusters (C1, C3) and intestinal eosinophils (Colon, SI). **(D)** Dendrogram of cross-tissue eosinophil similarity (1 − Spearman correlation). C3 clusters with blood eosinophils (r = 0.61), while C0, C2, C4, and C5 form a distinct adipose-specific branch.

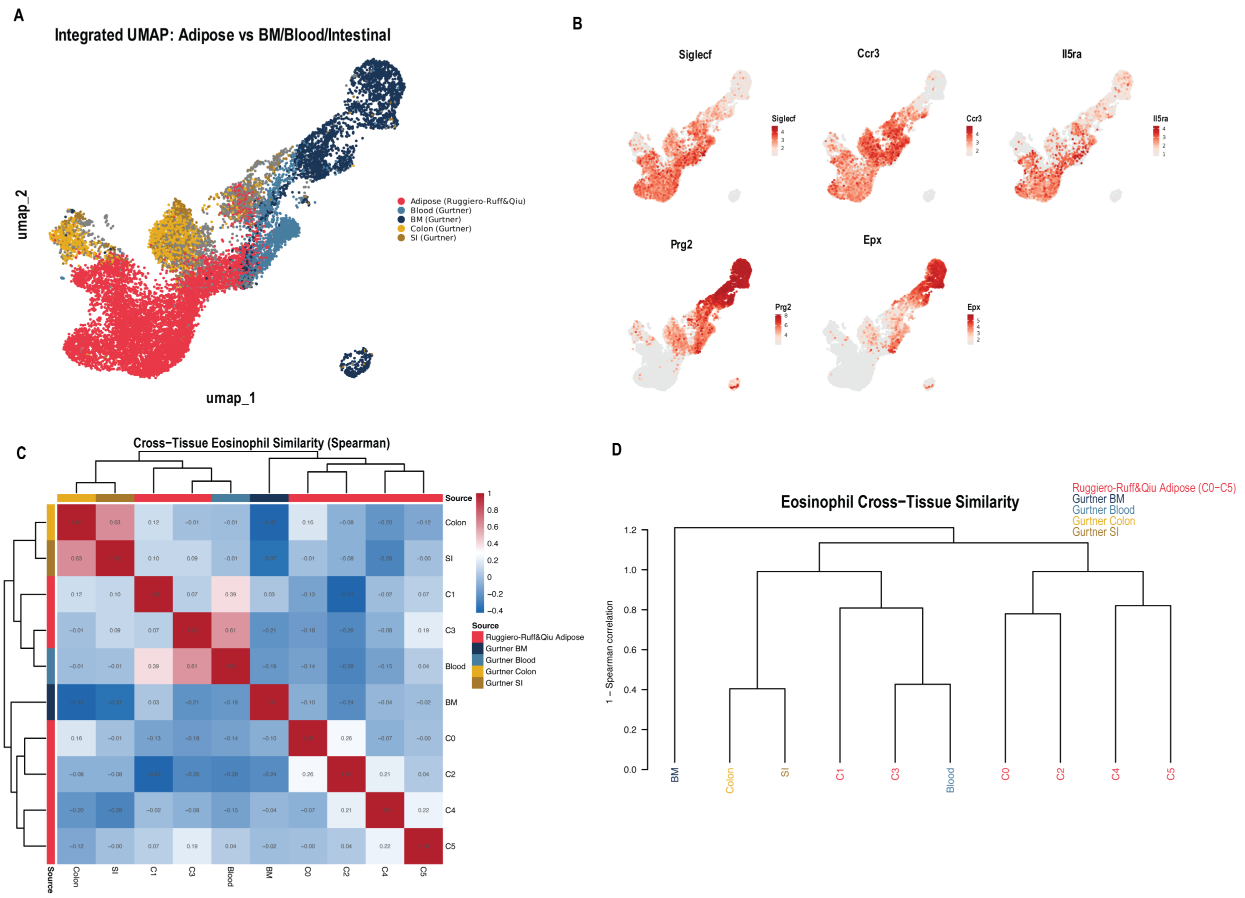

Figure S7

Figure S8. Macrophage cluster classification and validation. (A) Violin plots of module scores for M1, M2 Functional, and M2 Surface gene sets across the three macrophage clusters, confirming that each cluster scores highest on its own defining module. **(B)** UMAP of macrophages colored by cluster assignment (M1, M2 Functional, M2 Surface), showing spatially distinct populations. **(C)** Violin plots of *Retnla* (RELMα) expression by macrophage cluster and genotype (WT vs KO), confirming expression across all WT clusters and complete absence in KO. **(D)** Stacked bar plot of macrophage cluster composition across experimental conditions

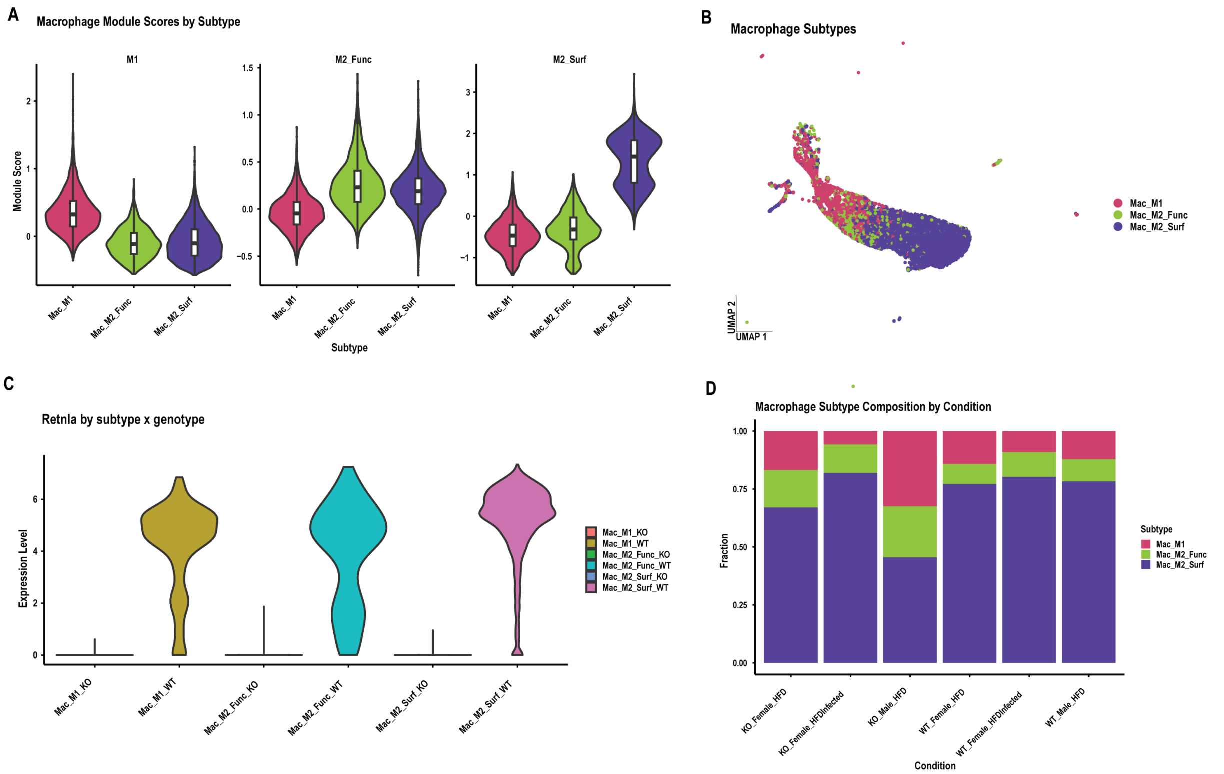

Figure S8

.

Figure S9. Regulon activity distributions across experimental groups. Violin plots showing AUCell score distributions for the top three cluster-enriched regulons per eosinophil cluster (C0–C5) across all six experimental conditions (WT♀, KO♀, WT♂, KO♂, WT♀ Infected, KO♀ Infected). Black dots represent per-sample mean AUCell scores (biological replicates). C0 regulons (*Zfp959*, *Mxd4*, *Fosb*) show strong male enrichment; C1 regulons (*Hoxc10*, *Hoxc6*, *Zfp174*) are female-enriched; C2 regulons (*Fos*, *Stat4*, *Zfp729b*) are predominantly active in male conditions. All regulons showed significant condition-dependent variation (Kruskal–Wallis $p < 10^{-4}$).

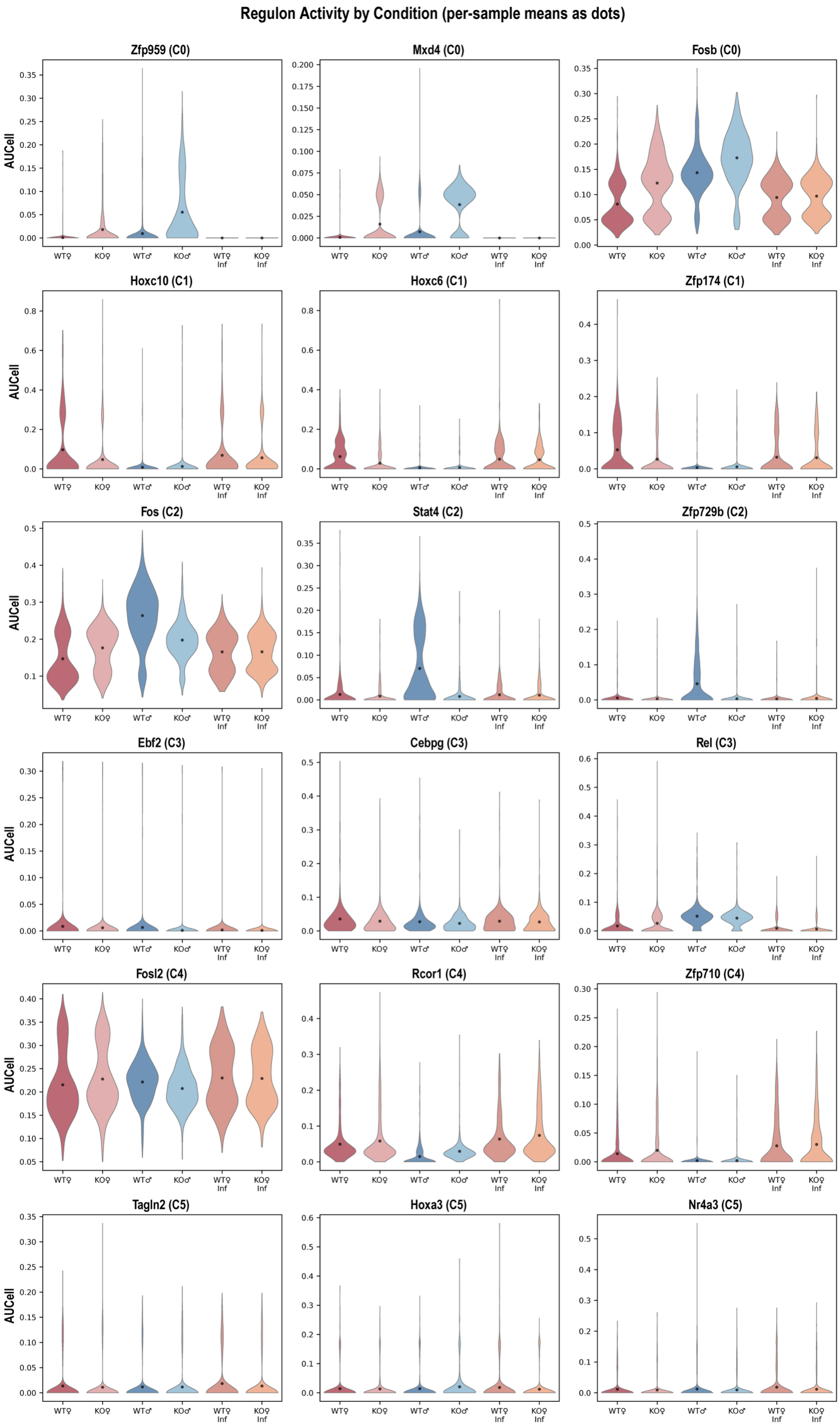
Regulon Activity by Condition (per-sample means as dots)
Zfp959 (C0)
Mxd4 (C0)
Fosb (C0)
Hoxc10 (C1)
Hoxc6 (C1)
Zfp174 (C1)
Fos (C2)
Stat4 (C2)
Zfp729b (C2)
Ebf2 (C3)
Cebpg (C3)
Rel (C3)
Fosl2 (C4)
Rcor1 (C4)
Zfp710 (C4)
Tagln2 (C5)
Hoxa3 (C5)
Nr4a3 (C5)
AUCell
WT♀
KO♀
WT♂
KO♂
WT♀ Inf
KO♀ Inf

Figure S9
